# Selection on the regulation of sympathetic nervous activity in humans and chimpanzees

**DOI:** 10.1371/journal.pgen.1007311

**Published:** 2018-04-19

**Authors:** Kang Seon Lee, Paramita Chatterjee, Eun-Young Choi, Min Kyung Sung, Jaeho Oh, Hyejung Won, Seong-Min Park, Youn-Jae Kim, Soojin V. Yi, Jung Kyoon Choi

**Affiliations:** 1 Department of Bio and Brain Engineering, KAIST, Daejeon, Republic of Korea; 2 School of Biology, Georgia Institute of Technology, Atlanta, Georgia, United States of America; 3 Specific Organs Cancer Branch, Research Institute, National Cancer Center, Ilsan, Gyeonggi, Republic of Korea; 4 Department of Neurology, University of California Los Angeles, Los Angeles, California, United States of America; University of Edinburgh, UNITED KINGDOM

## Abstract

Adrenergic α_2_C receptor (ADRA2C) is an inhibitory modulator of the sympathetic nervous system. Knockout mice for this gene show physiological and behavioural alterations that are associated with the fight-or-flight response. There is evidence of positive selection on the regulation of this gene during chicken domestication. Here, we find that the neuronal expression of ADRA2C is lower in human and chimpanzee than in other primates. On the basis of three-dimensional chromatin structure, we identified a *cis*-regulatory region whose DNA sequences have been significantly accelerated in human and chimpanzee. Active histone modification marks this region in rhesus macaque but not in human and chimpanzee; instead, repressive marks are enriched in various human brain samples. This region contains two neuron-restrictive silencer factor (NRSF) binding motifs, each of which harbours a polymorphism. Our genotyping and analysis of population genome data indicate that at both polymorphic sites, the derived allele has reached fixation in humans and chimpanzees but not in bonobos, whereas only the ancestral allele is present among macaques. Our CRISPR/Cas9 genome editing and reporter assays show that both derived nucleotides repress ADRA2C, most likely by increasing NRSF binding. In addition, we detected signatures of recent positive selection for lower neuronal ADRA2C expression in humans. Our findings indicate that there has been selective pressure for enhanced sympathetic nervous activity in the evolution of humans and chimpanzees.

## Author summary

Adrenergic α_2_C receptor (ADRA2C) is a regulator of the fight-or-flight response. It has been shown in mice that repression of this gene can result in relevant physiological and behavioral alterations. A strong selection signature in the genomes of domesticated chickens has been reported for this gene, suggesting that less aggression toward humans has been positively selected during chicken domestication. In this work, we analyze the genomes, transcriptomes, and epigenomes of a large number of humans and chimpanzees along with those of other primates to propose that repression of this gene has been positively selected in the evolution of humans and chimpanzees.

## Introduction

The sympathetic nervous system stimulates the fight-or-flight response of innervated target tissues by localized release of catecholamine neurotransmitters from the nerve terminals and by circulation of catecholamines released from the adrenal gland through the bloodstream. The adrenergic receptors are a class of G protein-coupled receptors that bind catecholamines, especially noradrenaline and adrenaline. α receptors have the subtypes α_1_ (G_q_ coupled receptor) and α_2_ (G_i_ coupled receptor). β receptors have the subtypes β_1_, β_2_ and β_3_. α2-adrenergic receptors are expressed not only on target tissues such as smooth muscles but also at sympathetic nerve terminals, where they function as inhibitory presynaptic autoreceptors that modulate the release of neurotransmitters.

In particular, the α_2C_ subtype (ADRA2C) modulates neurotransmission at lower levels of nerve activity in brain cortex [1]. Knockout mice for ADRA2C show more than a two-fold increase in circulating catecholamines [2] and behavioural alterations such as enhanced startle response, shortened attack latency, and diminished acoustic prepulse inhibition [3]. Multiple genes involved in the fight-or-flight response were identified as targets of selection during dog domestication [4]. In particular, one of the strongest selection signatures identified in the context of chicken domestication is located near ADRA2C [5]. Because there are no other known genes in the identified region, ADRA2C stands out as the most likely candidate. A follow-up study suggests that the target of selection may be noncoding regulatory regions that are distant from the gene body [6].

Genetic modifications in noncoding regulatory regions can be critical to human evolution. In their seminal work almost 40 years ago, King and Wilson [7] proposed a key role for regulatory modifications of noncoding DNA in shaping the evolution of our species. Indeed, the human genome contains noncoding DNA segments that are conserved in other species but show human-specific acceleration [8–12]. These accelerated elements were disproportionately found near genes involved in neuronal cell adhesion, indicating that noncoding changes in human evolution are associated with brain development and function [8].

Our incomplete knowledge of noncoding regions limits the functional interpretation of underlying DNA variants. Epigenomic signatures can mark the location of functional elements and provide systematic information on the spatiotemporal specificity of their regulatory activities [13–16]. Thus, the wealth of cell-type-specific human epigenomes provided by international consortia enable a systematic investigation of the regulatory mechanisms by which genetic variants affect phenotypes. For example, a majority of disease variants are located in DNase hypersensitive sites (DHSs) for the relevant cell types [13].

In this work, we sought to test whether noncoding regulatory regions of ADRA2C were under selection in humans by leveraging population genetics data and various epigenome data in neural cells or tissues. Humans and chimpanzees are the only primates that are known to frequently engage in warfare. Coalitionary attacks by chimpanzees on members of other groups resemble lethal intergroup raiding in humans. A recent study proposed that conspecific killing by chimpanzees is the result of adaptive strategies [17]. If intergroup aggression has been an adaptive or pervasive behaviour during the evolution of humans and chimpanzees, then the fight-or-flight response must have played a critical role in increasing fitness with constant exposure to lethal conflicts. Our study on the evolution of ADRA2C regulatory regions in humans will shed light on this hypothesis.

## Results

### Neuronal ADRA2C repression in human and chimpanzee

**Fig 1:**
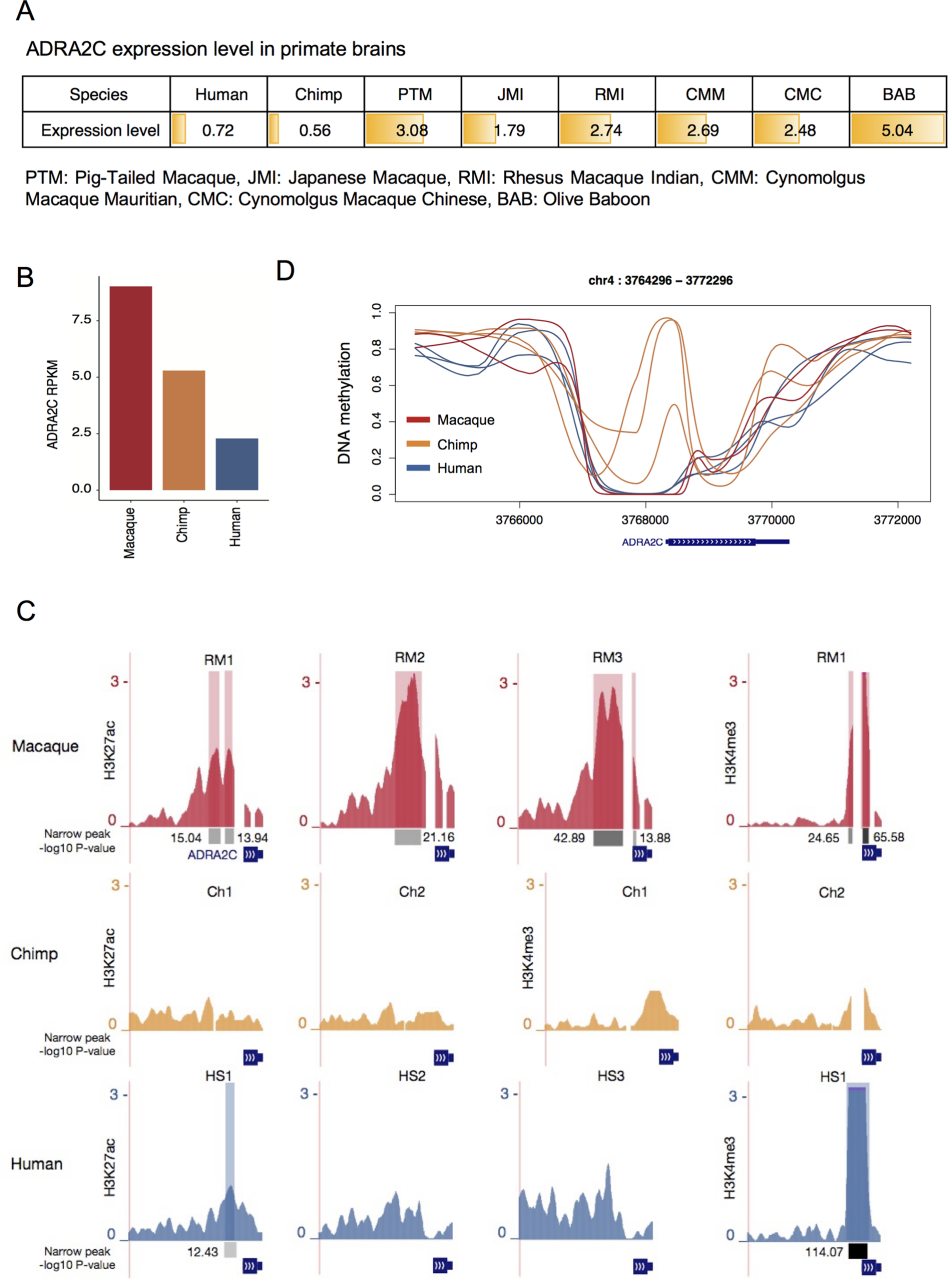
Neuronal ADRA2C expression in primates and epigenetic modification of the promoter in human, chimpanzee, and rhesus macaque. (A) Neuronal expression profile of ADRA2C in human and non-human primates. The expression levels were retrieved from the RNA-seq data of the Illumina Human BodyMap 2.0 and the Non-Human Primates Reference Transcriptome Resource. (B) Normalized RNA read counts for neuronal ADRA2C expression in human (blue), chimpanzee (orange), and rhesus macaque (red) from another dataset [18]. (C) Normalized ChIP-seq signals for H3K27ac and H3K4me3 at the promoter of ADRA2C in brain tissues from three rhesus macaque samples (RM1, RM2, and RM3), two chimpanzee samples (Ch1 and Ch2), and three *Homo sapiens* samples (HS1, HS2, and HS3) [19]. The ChIP-seq peaks (“narrow peaks”) and their statistical significance (−log10[P value]) are indicated. (D) DNA methylation levels at the ADRA2C locus in three human (blue), three chimpanzee (orange), and two rhesus macaque (red) brain samples [22]. CpG methylation levels were estimated from whole-genome bisulphite sequencing and were smoothed across the coordinates of the human reference genome.

We observe that the neuronal expression level of ADRA2C is lower in human and chimpanzee than in other primates (Fig 1A). Additional neural RNA-seq data for human, chimpanzee, and macaque [18] recapitulated this pattern (Fig 1B). We also examined chromatin immunoprecipitation-sequencing (ChIP-seq) data for H3K27ac and H3K4me3 in brain tissues from three *Homo sapiens* donors (HS1, HS2, and HS3), two chimpanzee donors (Ch1 and Ch2), and three rhesus macaque donors (RM1, RM2, and RM3) [19]. At the ADRA2C promoter, the two active histone marks were both enriched in the macaques but absent in the chimpanzees (Fig 1C). Promoter H3K27ac was low in the humans (Fig 1C), which is in agreement with the expression patterns. For comparison, we examined some genes that were consistently expressed in the three species. In contrast to ADRA2C, the promoter histone marks were present in all the species (S1 Fig).

However, promoter H3K4me3, which was examined in one human sample (HS1), was as high as in the macaque sample (RM1) (Fig 1C). Given the low expression level of ADRA2C in human, we hypothesized the presence of repressive histone modification. For example, bivalent domains, which are defined as regions marked by both activating (H3K4me3) and repressive (H3K27me3) modifications, can silence target genes in a poised state [20]. We examined ChIP-seq data for various histone modifications in human neuronal samples generated by the Roadmap Epigenomics project [21]. Indeed, the coexistence of H3K4me3 and H3K27me3 was observed in many samples (S2 Fig). Additionally, we examined DNA methylation maps for three human, three chimpanzee, and two macaque brains [22]. ADRA2C promoter methylation was particularly high in chimpanzees (Fig 1D). Taken together, ADRA2C is specifically down-regulated in human and chimpanzee while different repression mechanisms could act in the two species.

### Identification of a candidate *cis*-regulatory region

**Fig 2:**
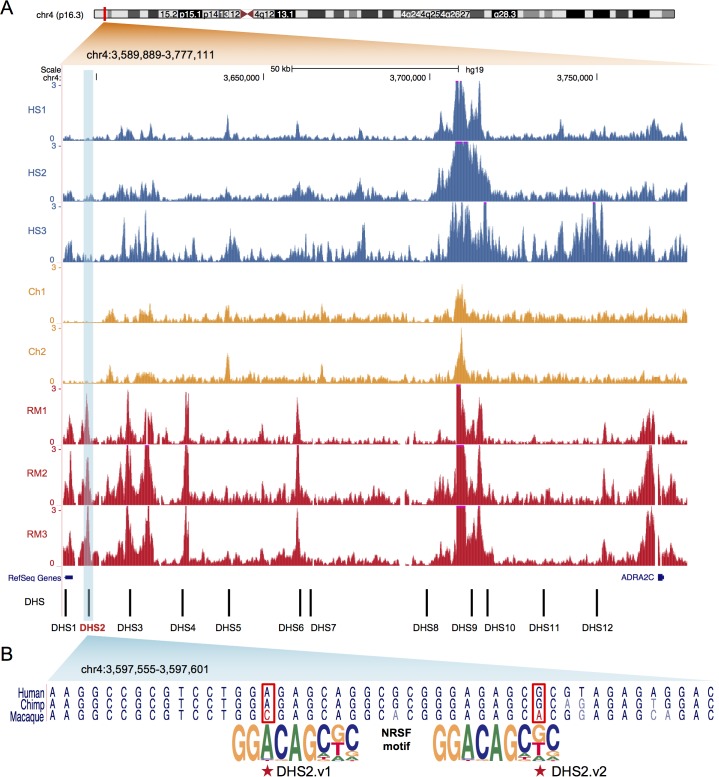
H3K27ac modifications and NRSF motifs of ADRA2C regulatory regions in human, chimpanzee, and rhesus macaque. (A) Normalized H3K27ac ChIP-seq signals across 12 distal regulatory regions (DHS1 ~ DHS12) responsible for ADRA2C in brain tissues from three *Homo sapiens* donors (HS1, HS2, and HS3), two chimpanzee donors (Ch1 and Ch2), and three rhesus macaque donors (RM1, RM2, and RM3) [19]. The genomic coordinates are based on hg19. For the DHS2 region, the “narrow peaks” were not called in HS1, HS2, HS3, Ch1, and Ch2. The significance (−log10[P value]) of the peak intensity for RM1, RM2, and RM3 was 18.02, 8.35, and 32.01, respectively. (B) Aligned reference genome sequences of human, chimpanzee, and rhesus macaque mapped to DHS2. Shown below are consensus motifs at two NRSF binding sites predicted with the human and chimpanzee sequences. The two motif SNPs that are identical between human and chimpanzee but different in rhesus macaque (DHS2.v1 and DHS2.v2) are highlighted.

We next attempted to pinpoint *cis*-regulatory regions that may contribute most to the human- and chimpanzee-specific repression of ADRA2C. First, using Hi-C data in human brain [23], we identified a topologically associating domain (TAD) that harbours ADRA2C. TADs represent three-dimensional chromosome structure that mediates most enhancer-promoter interactions within their boundaries [24]. Second, we identified 12 DHSs that were connected to the ADRA2C promoter within the TAD. Enhancer-to-promoter connections were identified by the correlation of the sequencing tag density between distal DHSs and promoter DHSs across cell types [25] (S3 Fig). Eleven of them overlapped neuronal DHSs (S1 Table). The genomic region spanning the 12 DHSs was similar to the chicken-domestication sweep in size and relative distance to the gene [6] (Fig 2A). Third, we examined histone modification patterns at these 12 DHSs. Five regions (DHS1, DHS2, DHS3, DHS4, and DHS6) carried H3K27ac in macaque brains only (Fig 2A), which recapitulated the promoter patterns (Fig 1C). Among the 5 differentially marked regions, DHS1, DHS2, and DHS4 were enriched for repressive histone modifications in various human neural samples, similar to the promoter DHS (S4 Fig). Fourth, we analyzed the brain Hi-C data [23] to find that DHS1, DHS2, and DHS3 are in physical contact with the ADRA2C promoter through chromatin structure (S5 Fig). Finally, we measured evolutionary acceleration in the lineage leading to human and chimpanzee for individual DHSs. On the basis of phyloP [26], DHS2 and DHS3 were determined to be the most accelerated sequences for the human-chimpanzee subtree (S2 Table). Altogether, DHS2 was singled out as the most likely candidate.

### Identification and functional validation of fixed regulatory variants

**Fig 3:**
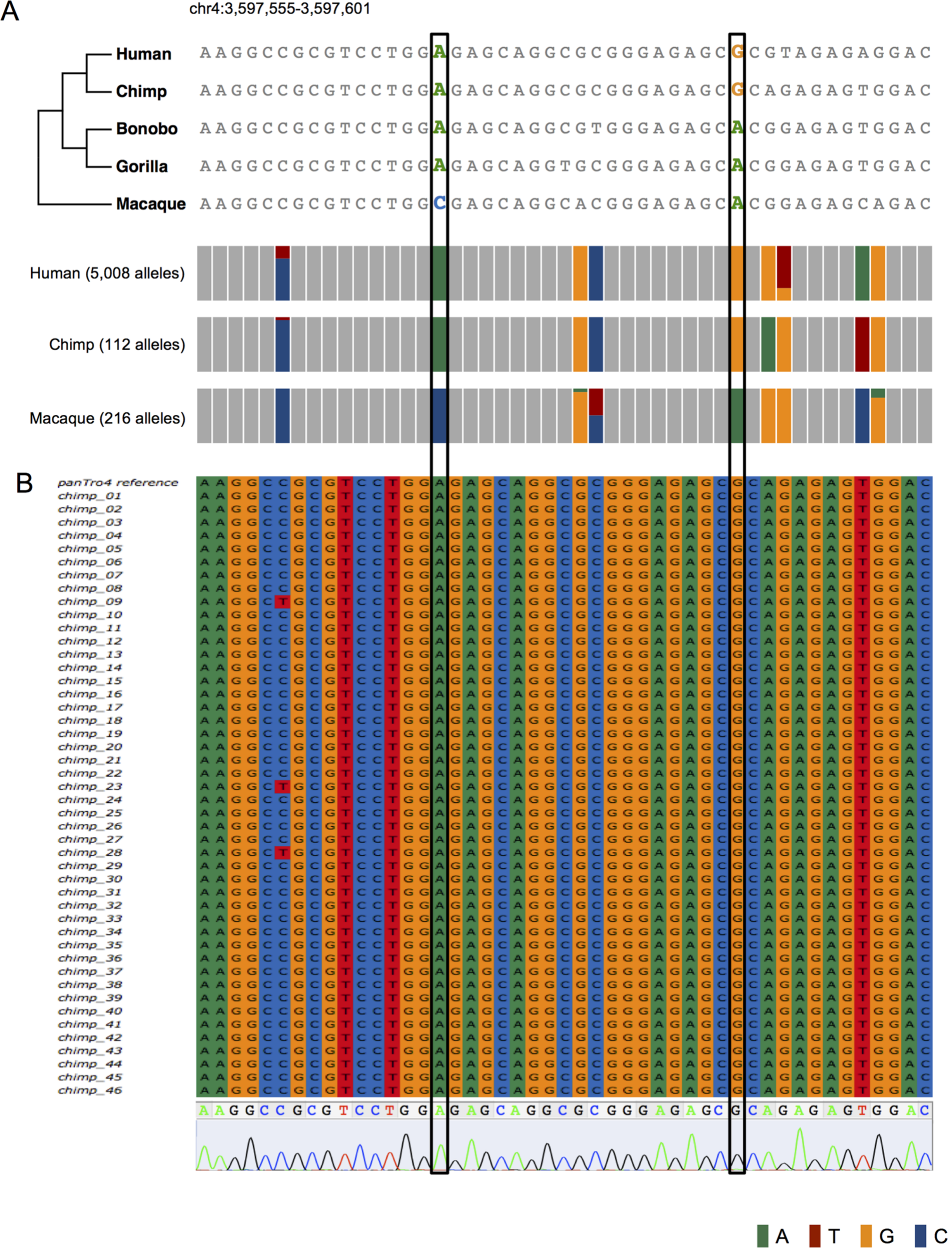
Genetic profile of DHS2.v1 and DHS2.v2. (A) Shown above is the alignment of reference sequences of human, chimpanzee, bonobo, gorilla, and macaque for the genomic region that encompasses the two NRSF motifs shown in Fig 2B. Shown below are the allele frequencies in the populations of humans, chimpanzees, and macaques at the single nucleotide polymorphic sites. The height of the coloured bars (A: green, C: blue, G: orange, and T: red) is proportional to the allele frequency. For chimpanzees, we merged 10 samples whose whole-genome sequencing data is available [28] and 46 samples for which we performed Sanger sequencing. DHS2.v1 and DHS2.v2 are marked by the black rectangles. (B) Alignment of Sanger sequencing reads for 46 unrelated chimpanzee samples (listed in S5 Table) mapped to panTro4. Shown below is a representative chromatogram. DHS2.v1 and DHS2.v2 are marked by the black rectangles.

We thus searched DHS2 for polymorphisms that fall in transcription factor binding motifs with the reference human and chimpanzee genomes harboring the derived allele and the reference macaque genome carrying the ancestral allele. Two variants, chr4:3597570 (DHS2.v1) and chr4:3597589 (DHS2.v2), met these conditions (Fig 2B). Intriguingly, each of these two variants was within a binding motif for neuron-restrictive silencer factor (NRSF) with the derived allele predicted to increase binding affinity (Fig 2B and S6 Fig). These sites corresponded to a region that shows high human-chimpanzee acceleration (S7 Fig). To profile genetic variation at the population level, we analyzed available genome sequences (2,504 human [27], 10 chimpanzee [28], and 108 macaque [29] samples) (Fig 3A and S3 and S4 Tables). Derived allele frequency in humans was 100% for DHS2.v1 and 99.98% for DHS2.v2. Similarly, all the chimpanzee chromosomes carried the derived allele at both variants. In contrast, all of the 108 macaque genomes were homozygous ancestral at both variants. We genotyped additional 46 unrelated chimpanzee samples (S5 Table) and observed only the derived allele at both sites (Fig 3B). The bonobo genome carried the ancestral allele at DHS2.v2, indicating that fixation has not been achieved among bonobos.

**Fig 4:**
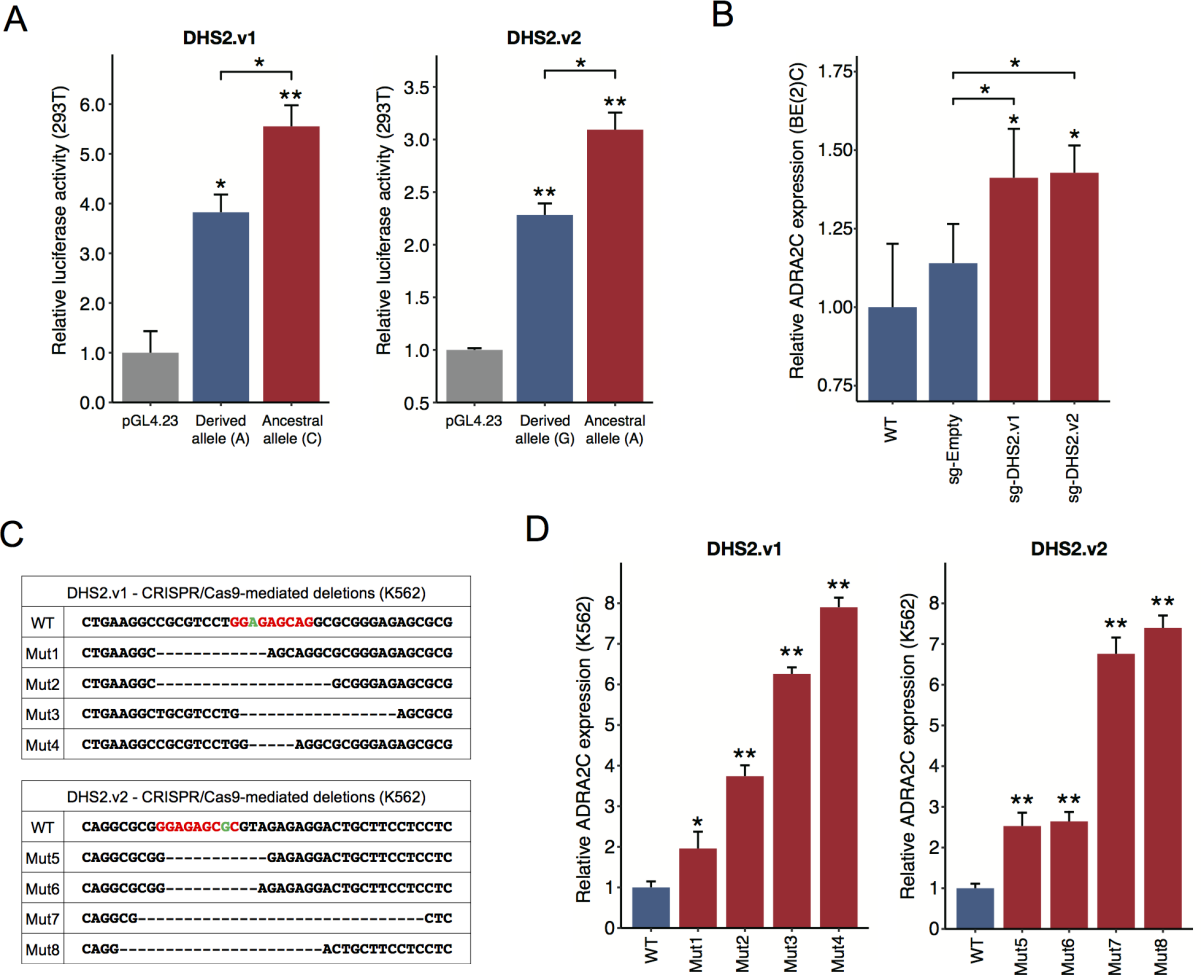
Regulatory activity of DHS2.v1 and DHS2.v2. (A) Results of luciferase reporter assays, which show the enhancer activity of the 300-bp sequences spanning each SNP with the ancestral allele (red) versus the derived allele (blue). Relative luciferase activity is shown in comparison to the transcriptional activity of the minimal promoter (pGL4.23). Three technical replicates were performed for each of three independent experiments. Data shown here is representative of the three experiments. P values were derived from two-tailed Student’s t-tests: *P ≤ 0.05, **P ≤ 0.005. Error bars, s.e.m. (B, D) Expression level of ADRA2C measured by qRT-PCR for the wild-type (derived) NRSF motif (blue) versus CRISPR/Cas9–mediated deletions (red). WT, wild-type BE(2)C; sg-Empty, no sgRNA; sg-DHS2.v1, targeted DHS2.v1 sgRNA; sg-DHS2.v2, targeted DHS2.v2 sgRNA (B) and WT, wild-type K562; Mut1~4, targeted DHS2.v1 sgRNA; Mut5~8, targeted DHS2.v2 sgRNA (D). Relative expression levels were computed by dividing by the wild-type measure. Three technical replicates were performed for each of three independent experiments. Data shown here is representative of the three experiments. P values were derived from two-tailed Student’s t-tests: *P ≤ 0.05, **P ≤ 0.005. Error bars, s.e.m. (C) Sanger sequencing results of individual clones that represent NRSF binding site mutations. The NRSF motifs are shown in red, and the two targeted variants (DHS2.v1 and DHS2.v2) are marked in green.

Both derived alleles were predicted to increase binding affinity for NRSF (Fig 2B and S6 Fig). According to our reporter assays, the genomic regions encompassing these variants possess enhancer activity, which is significantly lower with the derived allele than with the ancestral allele for both DHS2.v1 and DHS2.v2 (Fig 4A). To test the effect of the NRSF motifs on ADRA2C expression in the cellular chromatin environment, we designed a guide RNA for CRISPR/Cas9 genome editing with the aim of introducing the deletion of each motif. In neuronal cells, increased ADRA2C expression was observed in a population of transfected cells for both DHS2.v1 and DHS2.v2 (Fig 4B). We sought to isolate individual deletion clones using K562 cells. For each variant, four clones were successfully identified and their deletion breakpoints were determined by Sanger sequencing (Fig 4C and S8 Fig). All of the isolated clones consistently showed a significant overexpression of ADRA2C (Fig 4D).

### Signatures of recent selection for low ADRA2C expression in humans

The derived sequences are fixed or near-fixed at both sites in the present-day human population. Although more chimpanzee samples must be investigated to confirm fixation, the derived nucleotides are undoubtedly the major alleles in the population. We observed sequence acceleration in the lineage of human and chimpanzee (S2 Table). If the increased expression of ADRA2C was selected, we may also observe signatures of recent selection near the fixed sites.

**Fig 5:**
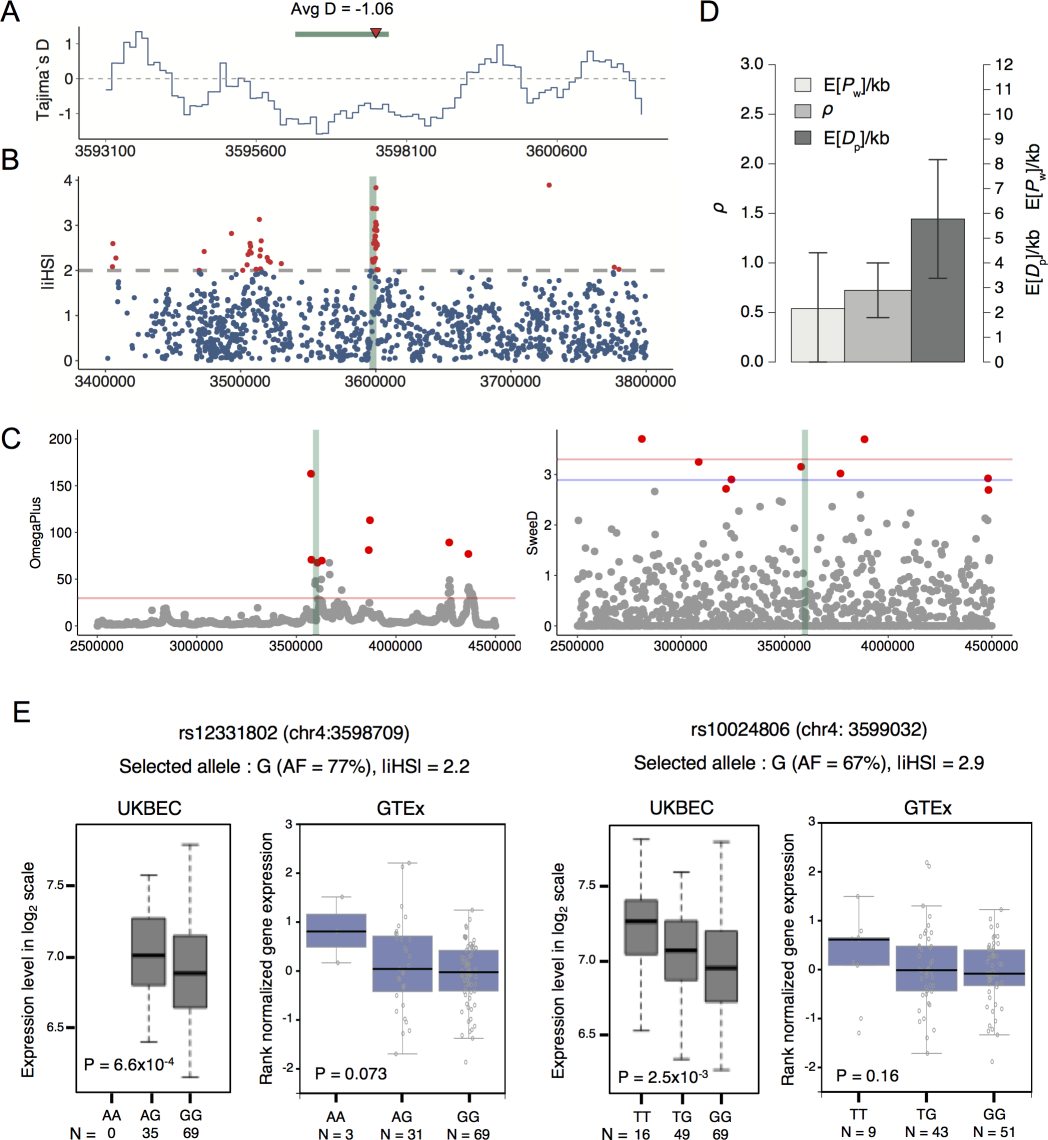
Evidence of positive selection on ADRA2C regulation in humans. (A) Tajima’s D for the DHS2-flanking region in humans. D was calculated for the +/- 4.5 kb region using a 1-kb window with a step size of 100 bp. The green bar is the neuronal DHS that encompasses the DHS2 SNPs (DHS2.v1 and DHS2.v2), whose location is marked by the red arrowhead. The average D for the neuronal DHS is shown. (B) iHS [33] calculated for the genomic region spanning the DHS2 locus by using a bioinformatics workflow for detecting signatures [61]. The location of DHS2 is marked by the green shade at the center. (C) Results of a compound test based on the CLR and ω statistic. We used SweeD [56] for the CLR test and OmegaPlus [58] for the ω statistic. The red and blue horizontal lines indicate cutoffs at P = 0.05 and P = 0.1, respectively, as obtained from a neutral hypothesis model. The outlier (top 1%) bins of the given region are marked by the red dots. The location of DHS2 is marked by the green shade at the center. (D) Significant positive selection on the 12 human regulatory sequences of ADRA2C (DHS1~DHS12). We applied INSIGHT [37,38] to infer selection on the collection of the human sequences from patterns of polymorphism and divergence with chimpanzee as the outgroup. *D*_p_ indicates the number of divergences driven by positive selection and is used as a measure of positive selection. *P*_w_ indicates the number of polymorphisms under weak negative selection. *ρ* is the fraction of sites under selection in general. Expected values for *D*_p_ and *P*_w_, E[*D*_p_] and E[*P*_w_], were divided by the total number of nucleotide sites considered in kilobases. (E) ADRA2C expression according to the genotype of rs12331802 and rs10024806. Shown above the plots are the selected allele determined by the iHS method, the allele frequency (AF) of the selected allele, and the iHS score. eQTL data from the UK Brain Expression Consortium (UKBEC) [40] and the Genotype-Tissue Expression (GTEx) project [39] were used.

When positive selection increases the frequency of a favoured allele, neighbouring neutral sequences are swept through the population along with the selected variant. This process causes a decrease in the level of genetic diversity, skew of the site frequency spectrum, and an excess of linkage disequilibrium (LD) [30]. We tested these three aspects for the DHS2 locus. First, low levels of nucleotide diversity (π) and negative Tajima’s D values [31] were observed (Fig 5A and S9 and S10 Figs). However, the Tajima’s D signals were not strong enough to support positive selection against genetic or statistical sampling bias. We computed the integrated haplotype score (iHS), which is a measure of the amount of extended haplotype homozygosity [32,33]. Strong iHS signals were observed at the DHS2 locus in the human population (Fig 5B). The details of the DHS2 iHS results (S11 Fig) suggest that chr4:3597632 is the candidate variant subjected to selection. This variant was assigned the greatest iHS score in this LD block, and the human major allele and chimpanzee reference allele were identical (S11 Fig). Similar to Tajima’s D, the composite likelihood ratio (CLR) statistic tests bias in the frequency spectrum [34]. Combining the CLR with an LD-based ω statistic [35] has been shown to increase the power to detect positive selection [36]. The compound test for the CLR and ω statistic supported positive selection on the target region (Fig 5C). It is possible that variants in other regulatory regions are also under selection. Indeed, a probabilistic method for testing recent selection on a collection of short interspersed noncoding elements [37,38] indicated significant positive selection on the 12 DHS regions in humans (Fig 5D). It is notable that most of the DHSs carry repressive marks in many, if not all, human neural samples (S3 Fig).

We sought to functionally test some of the variants that may be under recent selection. Because the iHS method estimated the selection strength and determined the selected allele for individual variants, we used the high-scoring (|iHS| > 2) variants at the DHS2 locus for this purpose. We searched the Genotype-Tissue Expression (GTEx) [39] data portal and BRAINEAC from the UK Brain Expression Consortium (UKBEC) [40] for the association of the brain ADRA2C expression level with the genotypes of the high-scoring SNPs. The eQTL data were available for two adjacent SNPs that were approximately 1 kb away from DHS2. The selected alleles for both variants were associated with lower ADRA2C expression in human brains (Fig 5E). This trend was observed for both the UKBEC and GTEx, but was only significant in the UKBEC results (P < 0.05). eQTL mapping based on whole-genome sequences may reveal additional functional variants under selection. One of the two eQTL SNPs, namely rs12331802, was located in the LD block encompassing DHS2.

## Discussion

We examined a variety of transcriptomic, epigenomic, and population genomic data to identify selected regulatory variants that are responsible for human- and chimpanzee-specific repression of an inhibitory modulator of sympathetic nervous activity. It is unclear when the two variants arose in the population and reached near-fixation. One of them (DHS2.v2) might have arisen more recently and reached near-fixation only in humans and chimpanzees. The other variant (DHS2.v1) appears to be older considering that the bonobo and gorilla reference genomes carry the derived allele (Fig 3A). The derived allele frequency in the population of bonobos and gorillas remains to be investigated. However, derived alleles do not necessarily lead to gene repression. Other genetic factors that act in *cis* or *trans* must also be accounted for. For example, the same sequences at the two variants in human and chimpanzee appear to entail different repression mechanisms (H3K27me3 versus DNA methylation). In humans, we also identified two segregating variants that appear to reflect positive selection for lower neuronal ADRA2C expression. Similar population genetic and functional analyses for chimpanzees may shed light on how a different repression mechanism (H3K27me3 compared to DNA methylation) has evolved in humans. Regardless of the underlying mechanism, our results suggest that there has been selective pressure for enhanced sympathetic nervous activity during the evolution of humans as well as chimpanzees. Humans and chimpanzees are the only primates that are known to engage in regular lethal aggression among neighbouring groups, in contrast to their closest relatives, bonobos. A recent study proposed that conspecific killing by chimpanzees is more the result of adaptive strategies than the response to human disturbances [17]. This proposal could explain the evolutionary roots of warfare, which may be a pervasive feature throughout human history [41]. A recent study has suggested that there is a phylogenetic component in conspecific violence of humans [42]. It remains to be investigated whether intergroup aggression was a major factor that exerted this selective pressure.

## Materials and methods

### Transcriptome and epigenome data

RNA-seq gene expression profile of ADRA2C in the brain of human and non-human primates were retrieved from the Illumina Human BodyMap 2.0 and the Non-Human Primates Reference Transcriptome Resource (http://nhprtr.org/) via AceView [43]. Additionally, RNA-seq data for the brain samples of human, chimpanzee, and rhesus macaque [18] were examined. RPKM values for ADRA2C and its orthologues were compared. We also examined H3K27ac and H3K4me3 ChIP-seq data for the brain samples of human, chimpanzee, and rhesus macaque [19]. The genomic location and statistical significance of the pre-defined “narrow peaks” were obtained. The panTro4 or rheMac3 sequence reads were lifted over to hg19/GRCh37. As for human brain, we examined 7 histone modifications (H3K4me1, H3K4me3, H3K27me3, H3K9ac, H3K36me3, H3K9me3, and H3K27ac) in 11 brain samples (Fetal brain, Germinal matrix, Neurosphere ganglionic eminence-derived, Neurosphere cortex-derived, Substantia nigra, Mid frontal lobe, Inferior temporal lobe, Hippocampus middle, Cingulate gyrus, Anterior caudate, and Angular gyrus). These data were obtained from the Roadmap Epigenomics project (http://www.roadmapepigenomics.org). HOMER (http://homer.ucsd.edu/homer/ngs/index.html) was run with the “–style histone” option to identify histone modification peaks. Additionally, we obtained fetal brain DHSs from the Roadmap Epigenomics project and postnatal DHSs from the ENCODE project [44] (http://genome.ucsc.edu/ENCODE). To identify putative *cis*-regulatory regions of ADRA2C, we first sought to define a TAD on the basis of Hi-C contact maps for two layers from the developing human brain, the cortical and subcortical plate (CP) and the germinal zone (GZ) [23]. We identified a TAD in each lamina as previously described [23] and used the intersection of the CP TAD and GZ TAD. We then examined enhancer-promoter connections within the TAD. The correlation of the sequencing tag density between distal DHSs and proximal DHSs across different cell types [25] resulted in 12 enhancer-promoter pairs that achieved the correlation coefficient >= 0.7. For the cell-type DHS map of the promoter and 12 enhancers (S2 Fig), we combined DHS datasets from the ENCODE project and Roadmap Epigenomics Project, which encompassed 156 cell types. We generated a set of neural DHSs by merging DHSs in various neural cell lines and brain tissues, including BE(2)C, SKNSH-RA, SK-N-MC, NPC (H1 derived neuroprogenitor cells), NT2_D1, fetal brain, and fetal spinal cord. We obtained the DNA methylation data of three human, three chimpanzee, and two rhesus macaque brains generated by whole-genome bisulphite sequencing [22]. Methylation levels at individual CpG sites in the ADRA2C promoter were compared. We analyzed Hi-C contact profiles of the ADRA2C promoter as previously described [23]. Briefly, the statistical significance of chromatin interactions for the 10-kb bin containing the ADRA2C promoter was assessed using a background Hi-C interaction profile generated from random regions of the genome with matched GC content for gene promoters [23].

### Processing of genome sequencing data

We used the genome data of 10 unrelated chimpanzees [28] and 108 unrelated rhesus macaques [29]. Out of 133 samples that were reported to be sequenced, only 108 were available for download. The whole genome sequencing data were obtained from the Sequence Read Archive (SRA; https://www.ncbi.nlm.nih.gov/sra). The NCBI BioProject database accession numbers were PRJEB1357 for the chimpanzee data and PRJNA251548 for the macaque data. The sample lists are provided in S3 and S4 Tables. Sequence reads were aligned using the BWA [45] to the respective reference genomes (i.e., panTro4 and rheMac3). Duplicate reads were removed by using the Picard tools (http://broadinstitute.github.io/picard/). We used the GATK’s HaplotypeCaller [46] for genotyping calling. GATK Variant Filtration was performed to retain the sites in which the map quality (MQ) is >= 30, the Phred scaled probability that a polymorphism exists (QUAL) is >= 30, and the fraction of reads that cover the position whose MQ = 0 (MQ0/DP) is < 0.1. The panTro4 and rheMac3 VCF files were lifted over to hg19/GRCh37. We obtained the VCF files for the genome sequences of 2,504 present-day humans from the 1000 Genomes [27] Phase 3 analysis results (ftp://ftp.1000genomes.ebi.ac.uk/vol1/ftp/release/20130502/).

### Measuring human-chimpanzee acceleration

Lineage-specific evolutionary acceleration was estimated on the basis of the likelihood ratio test implemented by the PhyloP (Phylogenetic P-values) algorithm [26] of the PHAST package (http://compgen.bscb.cornell.edu/phast). This test compares the substitution rates of conserved regions between lineages of interest and the remainder of the tree. We applied phyloP for the multiple alignments of primate genome sequences. The multiple alignments in the maf format were obtained from the UCSC Genome Browser (http://hgdownload.soe.ucsc.edu/goldenPath/hg19/multiz46way/maf/). PhyloP was run with “--subtree hg19-panTro2”, “--method LRT”, and “--mode CONACC” as options. Primates.mod was used as the neutral model. With the “--features” option, each of the 12 regulatory regions was scored (S2 Table). In this case, we targeted the neuronal DHSs that overlapped the *cis*-regulatory regions of ADRA2C (i.e., DHS1~DHS12). Regions with “alt_subscale >1” were interpreted as having evolved faster in the human-chimpanzee subtree than the remaining part of the tree. The “--wig-scores” option was used for the base-by-base scores of the DHS2 locus plotted in S6 Fig.

### Genotyping of chimpanzee samples

For DHS2 genotyping, DNA samples from 46 unrelated chimpanzee individuals were obtained. The samples used are listed in the S5 Table. These samples include genomic DNA extracted from liver samples of 6 individuals obtained from the Yerkes National Primate Research Center, genomic DNA from 10 individuals purchased from the Coriell Institute of BioBank, and genomic DNA extracted from 34 cell lines purchased from the Coriell Institute of BioBank. Genomic DNA from liver tissues was extracted using the Qiagen DNeasy Blood and Tissue DNA extraction kit (Qiagen) according to the manufacturer’s instructions. Genomic DNA from cell cultures was extracted using the Qiagen Blood and Cell Culture DNA Mini Kit. The extracted DNA was re-suspended in TE buffer and quantified by Qubit. PCR primers were designed to target the DHS2 regulatory region of ADRA2C (631 bp). The PCR primers sequence are provided in the S6 Table. The PCR conditions were optimized and tested to confirm that the right and best PCR products were reproducible. The samples were amplified using 10x Thermo Fisher PCR buffer, 1.25 mM dNTP, 2 mM Mg^++^, 1 U of Taq polymerase enzyme (Thermo fisher), 10 μM of Forward and Reverse primers using the following PCR conditions: 95°C for 5 min; 94°C for 30 s; annealing temperature at 66°C for 25 s; and 35 cycles and final extension at 72°C for 10 mins. The amplified PCR products were purified using the QIAquick PCR purification Kit (Qiagen) and were sent for Sanger sequencing for the forward and reverse strands. Following these steps, we collected sequence information from 46 samples. The obtained nucleotide sequences were aligned by CLUSTALW using MEGA7 (Molecular Evolutionary Genetics Analysis Version 7.0) [47] (available at http://www.megasoftware.net). To reduce the possible effect of PCR artefacts, unique substitutions in single clones were ignored.

### Motif analysis

We searched the DHS2 sequences for transcription factor binding sites on the basis of the TRANSFAC [48–50] and JASPAR [51–54] databases by running FIMO [55] at the P value threshold of 10^-3^. Our motif search was performed for the reference human and chimpanzee genomes (hg19 and panTro4) that carried the derived allele and the reference macaque genome (rheMac3) that carried the ancestral allele. We detected two variants, chr4:3597570 (DHS2.v1) and chr4:3597589 (DHS2.v2), each within a binding motif for NRSF, with the derived allele predicted to increase its binding affinity.

### Cell lines

In this study, we used three cell lines, namely, BE(2)C (CRL-2268), K562 (CCL-243), and 293T (CRL-3216), which were obtained from American Type Culture Collection (ATCC). These cell lines were cultured in complete DMEM (BE(2)C, 293T) or RPMI-1640 medium (K562) (Life Technologies) supplemented with 10% fetal bovine serum (Life Technologies) and 1% penicillin-streptomycin (Life Technologies). The cells were maintained at 37°C in a humidified chamber supplemented with 5% CO_2_.

### Luciferase reporter assays

300-bp wild-type (derived) and mutant (ancestral) sequences centered on DHS2.v1 or DHS2.v2 were synthesized with a restriction enzyme site for Kpn I or Nhe I at each end. These sequences were then inserted into the pGL4.23 luciferase reporter vector. Luciferase assays were performed by using the Dual-Luciferase reporter assay system (Promega) according to the manufacturer’s instructions. One microgram of the wild-type (derived), mutant (ancestral), or minimal promoter construct, along with 0.1 μg of Renilla luciferase vector (Promega), were transfected into 293T cells plated at a density of 5.0 x 10^4^ cells per well in 24-well plates. For transfection, the cells were incubated with Lipofectamin 2000 in Opti-MEM medium for 4 h. After 48 h of transfection, the cells were extracted, and luciferase activity was measured using a VICTOR Light luminometer (PerkinElmer). The ratio of firefly to Renilla luciferase activity was obtained. Relative luciferase activity was obtained by dividing by the measures for the minimal promoter construct. Measurements were made in triplicate wells for each of three independent experiments.

### CRISPR/Cas9-mediated genome editing

Two single guide RNA (sgRNA) each flanking the two NRSF motifs (chr4:3597567-3597576 and chr4:3597582-3597590) were designed by RGENs (http://www.rgenome.net). These sgRNAs were cloned into pSpCas9(BB)-2A-GFP (PX458, Addgene, #48138) and pSpCas9(BB)-2A-Puro (PX459, Addgene, #62988). The pSpCas9(BB)-2A-Puro vector with sgRNA were transfected into BE(2)C cells using the Lipofectamine 3000 transfection reagent (Life Technologies) in Opti-MEM medium for 6 h. For selection, the transfected cells were cultured in media with 2 μg/ml puromycin (Life Technologies) for 48 h. The pSpCas9(BB)-2A-GFP vector with sgRNA was transfected into K562 cells using the Neon Transfection System Kit (Thermo Fisher Scientific). After 48 h, transfected GFP-positive cells were individually isolated. The single cell clones were individually cultured into single wells for two weeks. To verify the deletion of the NRSF motif region, genomic DNA was extracted from the transfected cells by the DNeasy Blood and Tissue Kit (Qiagen) and amplified by PCR using Hifi Hot Sart (KAPA). The obtained PCR products were sequenced in both forward and reverse orientations by Sanger sequencing. RNA was extracted by the RNeasy Plus mini kit (QIAGEN), and cDNA was synthesized from total RNA using SuperScript IV VILO Master Mix (Invitrogen). qRT-PCR was performed using the SYBR Green PCR Master Mix (Applied Biosystems) on the QuantStudio 5 Real-Time PCR System (Applied Biosystems). The ADRA2C expression levels were measured by qRT–PCR and normalized to the GAPDH levels. Each of three independent experiments was performed with three technical replicates. All of the PCR primers and CRISPR sgRNA sequences are provided in S6 Table.

### Tests for positive selection

To test selective sweep for the DHS2 locus, we used the 2,504 human genomes [27]. The Tajima’s D statistic is estimated based on the difference between the mean number of pairwise nucleotide differences and the number of segregating sites. Under neutrality, these two measures have equal expectations and Tajima’s D will be close to 0. Positive values of Tajima’s D suggest an excess of common variation in a region, which can be consistent with balancing selection. Negative values of Tajima’s D indicate an excess of rare variation, which is consistent with positive selection. The statistic is calculated with the following parameters: *n*, the number of chromosomes; *S_n_*, the number of polymorphic sites observed; and *p_i_*, the major allele frequency of the *i*th SNP. With these parameters, Tajima’s D was obtained as

$$D=\frac{\pi -\theta_{s}}{\sqrt{Var(\pi -\theta_{s})}},$$ (1)

where

$$\pi =\frac{n}{n-1}\sum_{i=1}^{S_{n}}2p_{i}(1-p_{i})$$ (2)

and

$$\theta_{s}=\frac{S_{n}}{\sum_{i=1}^{n-1}\frac{1}{i}}.$$ (3)

We calculated the nucleotide diversity and Tajima’s D [31] for the DHS2 locus by running a 1-kb window with a step size of 100 bp. To compute the average nucleotide diversity for each window, we divided the overall nucleotide diversity, *π*, defined above by the total number of bases within the window because π = 0 at invariant sites. We also tested 10 chimpanzee [28] and 108 macaque [29] genomes. Additionally, we used our 337-bp sequences of the 46 chimpanzee samples. Based on the UCSC liftOver tool, we identified chimpanzee (panTro4) and macaque (rheMac3) positions that are orthologous to human (hg19). CLR is calculated by multiplying the probabilities of all polymorphic sites of a given region, which makes it possible to estimate the strength and location of a selective sweep [34]. This method returns a likelihood of a complete sweep compared to a population that neutrally evolves. Combining a composite likelihood method with an LD-based test based on the ω statistic [35] was shown to increase the power to detect positive selection and reduce the number of false positives [36]. Currently, SweeD [56] is regarded as the most advanced CLR-based test [57]. The ω statistic can be implemented by OmegaPlus [58]. To detect the common outliers of the SweeD and OmegaPlus analyses, we ran R scripts available at http://pop-gen.eu/wordpress/server-for-selective-sweep-detection for the 2Mb genomic region that spanned DHS2. Significance was estimated based on neutral models generated by msHOT [59], which is a modified version of Hudson’s ms simulator [60]. iHS was developed [33] on the basis of the EHH (extended haplotype homozygosity) statistic [32]. The EHH measures the decay of identity, as a function of distance, of haplotypes that carry a specified core allele. Extreme (|iHS| > 2) iHS scores indicate that haplotypes with the core allele are significantly longer than those with background alleles. We used a bioinformatics workflow [61] to compute iHS and determine the selected allele for individual variants. To collectively test selection on the sequences of the 12 DHSs, we applied INSIGHT (http://compgen.cshl.edu/INSIGHT/), which is a probabilistic method for inferring selection signatures from a collection of short interspersed genomic elements by contrasting patterns of polymorphism and divergence with patterns observed in flanking neutral sites [37,38].

### eQTL analysis

We examined two eQTL databases, the Genotype-Tissue Expression (GTEx) [39] data portal (https://www.gtexportal.org) and BRAINEAC from the UK Brain Expression Consortium (UKBEC) (http://www.braineac.org) [40]. We searched the databases for the SNPs with an extreme (|iHS| > 2) iHS score at the DHS2 locus, corresponding to the red dots overlapping the green shade at the center of the plot of Fig 5B. The eQTL data were available for two of them, that is, rs12331802 (chr4:3598709) and rs10024806 (chr4:3599032). We surveyed the association of the genotypes of these two polymorphisms with ADRA2C expression levels in different brain subregions. The most significant association was selected.

## Supporting information

S1 Fig Histone modification patterns of illustrative genes whose expression level was consistent among human, chimpanzee, and macaque. The intensities of the promoter histone modification peaks (-log10[P values] of the “narrow peaks”) of these genes were compared with those of ADRA2C in the upper right heatmaps. For illustration, the expression and histone modification patterns of BRF2 were compared with those of ADRA2C as shown in Fig 1B and 1C.
(PDF)

S2 Fig Characterization of the ADRA2C promoter as a bivalent chromatin domain in various human brain tissues. ChIP-seq signals for activating histone modifications (H3K4me3) and repressive histone modifications (H3K27me3) in 10 brain tissues (Angular gyrus, Anterior caudate, Germinal matrix, Hippocampus middle, Inferior temporal lobe, Mid frontal lobe, Substantia nigra, Fetal brain, Neurosphere cortex derived, and Neurosphere ganglionic eminence derived) were from the Roadmap Epigenomics project. The correlation plots were drawn between the two marks for 20-bp bins across the region +/- 1kb of the tss. ChIP-seq signals were assigned to each bin.
(PDF)

S3 Fig DHS patterns of the 12 distal regulatory regions (DHS1~DHS12) and ADRA2C promoter. The 12 cis-regulatory regions were identified based on the correlation of the sequencing tag density between distal DHSs and proximal DHSs across different cell types within a human brain TAD. Shown here is a cell-type-specific DHS map for these 12 regions and ADRA2C promoter (columns). We combined DHS datasets from the ENCODE project and Roadmap Epigenomics Project, covering 156 cell types (rows).
(PDF)

S4 Fig (A) Histone modification patterns of the 12 ADRA2C regulatory regions. ChIP-seq data for activating histone modifications (H3K4me1, H3K4me3, H3K9ac, H3K27ac, and H3K36me3) and repressive histone modifications (H3K9me3 and H3K27me3) in 11 brain tissues (Fetal brain, Germinal matrix, Neurosphere ganglionic eminence derived, Neurosphere cortex derived, Substantia nigra, Mid frontal lobe, Inferior temporal lobe, Hippocampus middle, Cingulate gyrus, Anterior caudate, and Angular gyrus) were obtained from the Roadmap Epigenomics project. Peak finding was performed by using HOMER. We obtained 14 fetal brain DHS datasets from the Roadmap Epigenomics project and 13 postnatal brain DHS datasets from the ENCODE project. They were merged into five categories (fetal brain, fetal spinal cord, neural progenitor cells, adult brain, and infant brain). The 12 ADRA2C regulatory regions were mapped to the histone modification peaks or DHSs. (B) ChIP-seq signals for H3K27me3 and H3K9me3 near DHS2 in the brain tissues shown in (A).
(PDF)

S5 Fig Chromatin interaction of the ADRA2C promoter in human brain. Statistical significance of its Hi-C interactions with the 12 distal regulatory regions (red and grey ticks below the chromosome ideogram and genome axis on the top) was measured for 10-kb bins using a background Hi-C interaction profile generated from random regions of the genome with matched GC content for gene promoters and was plotted as −log10[P value]. The green line is for the cortical and subcortical plate (CP) and the orange color line is for the germinal zone (GZ). The ADRA2C gene is marked in blue. The grey dotted line marks FDR = 0.01.
(PDF)

S6 Fig Comparison of NRSF binding affinity between the derived allele and ancestral allele of DHS2.v1 and DHS2.v2. (A) Motif score derived from FIMO. (B) −log10[P value] of the FIMO motif score.
(PDF)

S7 Fig Evolutionary acceleration of DHS2. The location of the two NRSF motifs containing DHS2.v1 and DHS2.v2 is marked. The “Neuron DHS” track displays a union of DHSs in various neural cell lines and brain tissues, including BE(2)C, SKNSH-RA, SK-N-MC, NPC (H1 derived neuroprogenitor cells), NT2_D1, fetal brain, and fetal spinal cord, obtained from the ENCODE project and Roadmap Epigenomics project. We applied phyloP for the multiple alignments of primate genome sequences with “--subtree hg19-panTro2”, “--method LRT”, “--mode CONACC”, and “--wig-scores” as options. Negative values (red lines) indicate acceleration.
(PDF)

S8 Fig Deletion breakpoints generated by CRISPR/Cas9 for the four DHS2.v1 clones (upper) and the four DHS2.v2 clones (lower). The sequences of each PCR fragment were used to characterize the deletions. The NRSF binding motif, which was targeted for deletion, is marked in red.
(PDF)

S9 Fig Tajima’s D for the DHS2 locus in chimpanzees (upper) and macaques (lower). D was calculated for the +/- 4.5 kb region using a 1-kb window with a step size of 100 bp. Negative values imply positive selection. The green bar is a neuronal DHS that encompasses the DHS2 SNPs (DHS2.v1 and DHS2.v2), whose location is marked by the red arrowhead. The blue dot in the chimpanzee plot indicates D for the 337-bp flanking sequences of the 46 chimpanzee samples. The average D for the neuronal DHS is shown.
(PDF)

S10 Fig Nucleotide diversity for the DHS2 locus in humans (top), chimpanzees (middle), and macaques (bottom) whose whole-genome sequences were available. π was calculated for the +/- 4.5 kb region using a 1-kb window with a step size of 100 bp. Low nucleotide diversity is associated with positive selection. The green bar is a neuronal DHS that encompasses the DHS2 SNPs (DHS2.v1 and DHS2.v2), whose location is marked by the red arrowhead. The grey dotted horizontal lines mark the average diversity of the region.
(PDF)

S11 Fig The details of the iHS results on the variants within the LD block containing DHS2. A positive (or negative) iHS score means that haplotypes on the ancestral (or derived) allele background are longer compared to the derived (or ancestral) allele background. The last column of the below table shows the selected allele inferred according to the sign of the iHS score (A for positive iHS and D for negative iHS). The candidate variant for selection was highlighted. A possible scenario is that the ancestral haplotype acquired the derived sequence, T, before human-chimpanzee divergence at this position, which has been selected in the two species. This may be why the iHS results suggest selection for the alleles carried on the ancestral haplotype.
(PDF)

S1 Table Chromosomal coordinates of ADRA2C regulatory regions identified based on DNase I hypersensitivity. (PDF)

S2 Table Conservation or acceleration of ADRA2C regulatory sequences as estimated based on the likelihood ratio test of phyloP for the subtree of human and chimpanzee. (PDF)

S3 Table List of 10 unrelated chimpanzee samples whose genome data was used in this work. (PDF)

S4 Table List of 108 unrelated rhesus macaque samples whole genome data was used in this work. (PDF)

S5 Table List of 46 genotyped chimpanzee samples. (PDF)

S6 Table CRISPR/Cas9 single guide RNA (sgRNA) sequences and PCR primers used in this study. (PDF)
