# Supplementary figures and images for: Selection on the regulation of sympathetic nervous activity in humans and chimpanzees
Source: PLoS Genet. 2018 Apr 19;14(4):e1007311. doi: 10.1371/journal.pgen.1007311 (PMC5908061; doi:10.1371/journal.pgen.1007311)

Supplementary Fig. 1

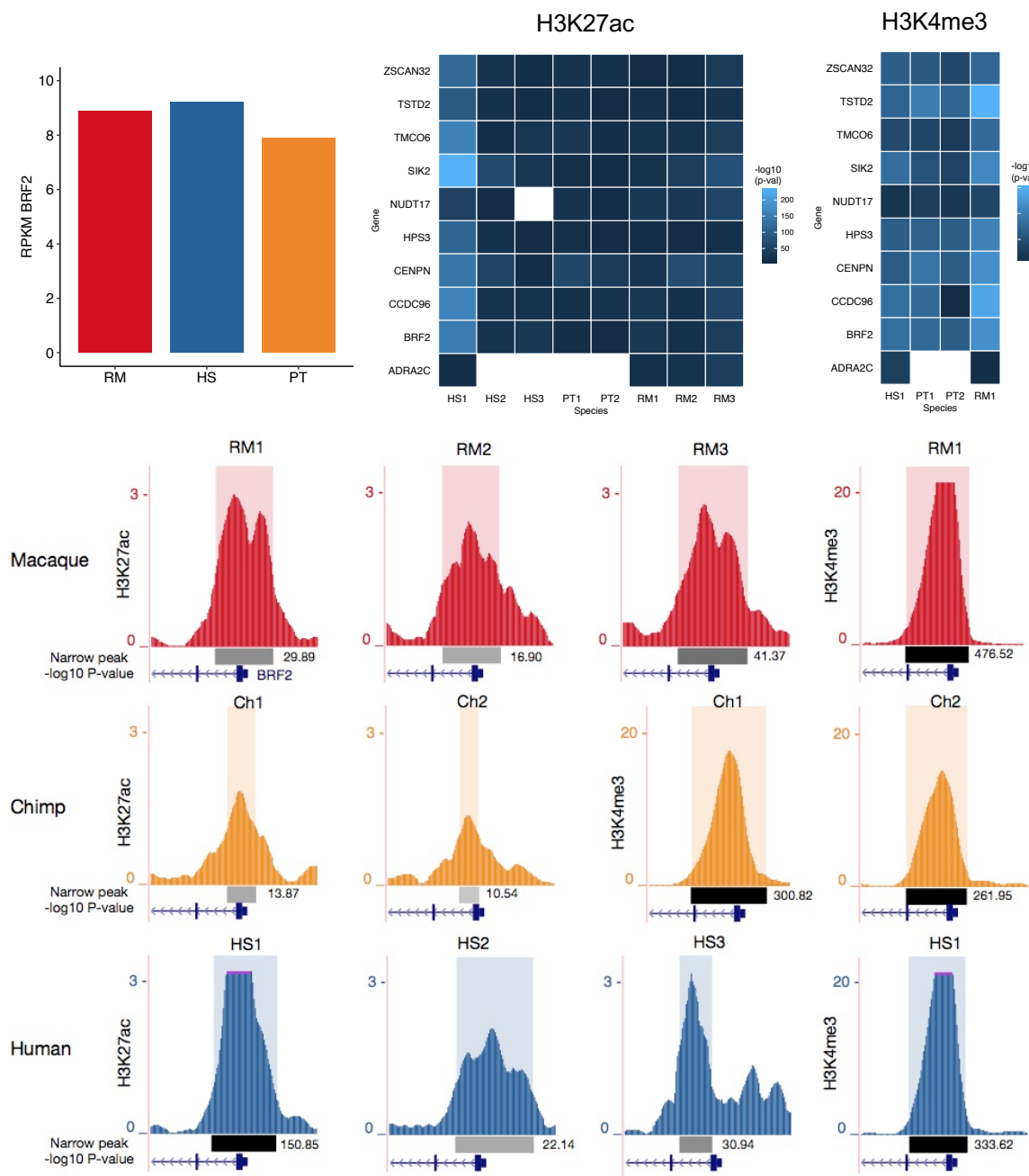

Supplement: S1 Fig — The intensities of the promoter histone modification peaks (-log10[P values] of the “narrow peaks”) of these genes were compared with those of ADRA2C in the upper right heatmaps. For illustration, the expression and histone modification patterns of BRF2 were compared with those of ADRA2C as shown in Fig 1B and 1C. (PDF) [file pgen.1007311.s001.pdf]

Supplementary Fig. 2

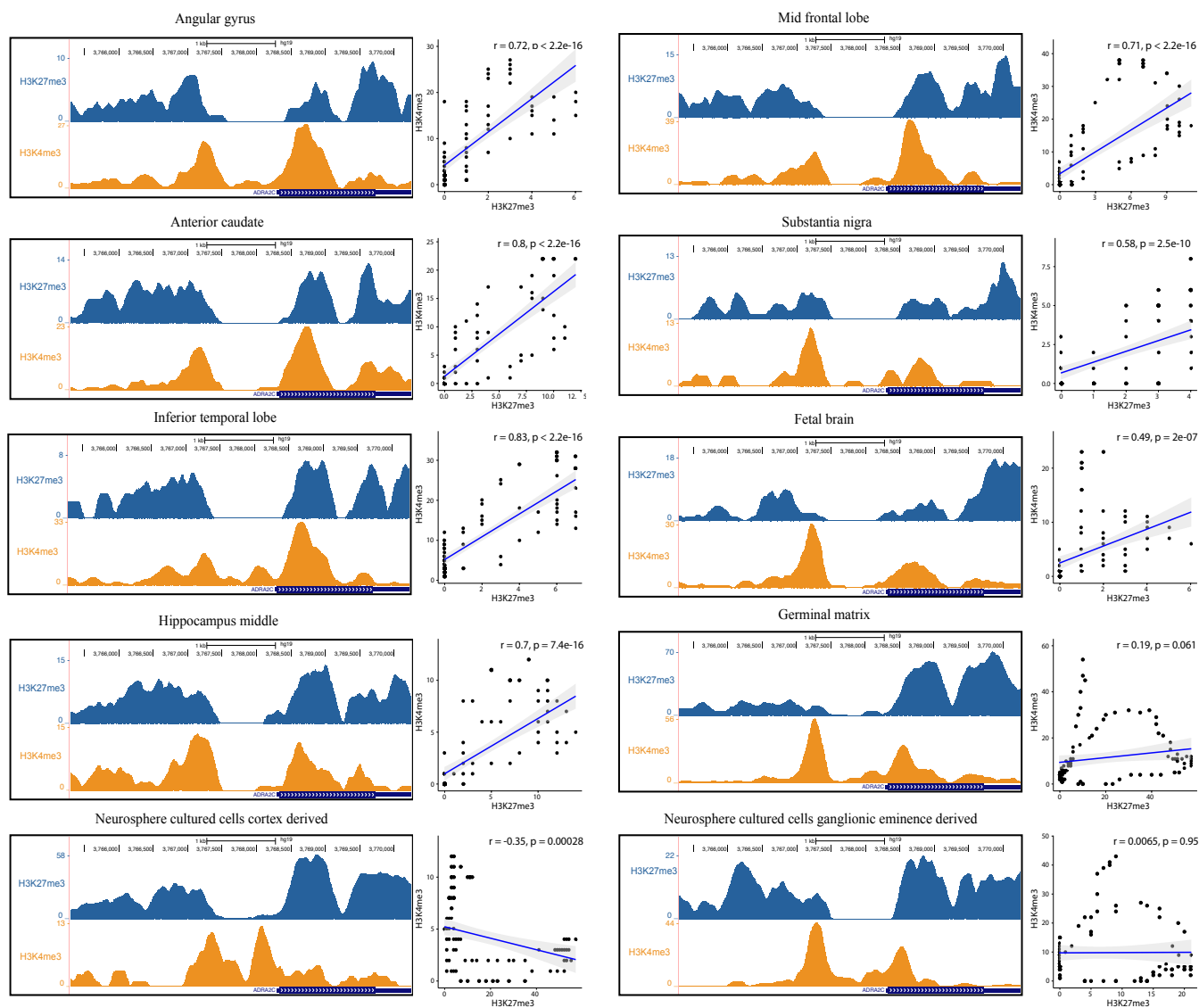

Supplement: S2 Fig — ChIP-seq signals for activating histone modifications (H3K4me3) and repressive histone modifications (H3K27me3) in 10 brain tissues (Angular gyrus, Anterior caudate, Germinal matrix, Hippocampus middle, Inferior temporal lobe, Mid frontal lobe, Substantia nigra, Fetal brain, Neurosphere cortex derived, and Neurosphere ganglionic eminence derived) were from the Roadmap Epigenomics project. The correlation plots were drawn between the two marks for 20-bp bins across the region +/- 1kb of the tss. ChIP-seq signals were assigned to each bin. (PDF) [file pgen.1007311.s002.pdf]

## Supplementary Fig. 3

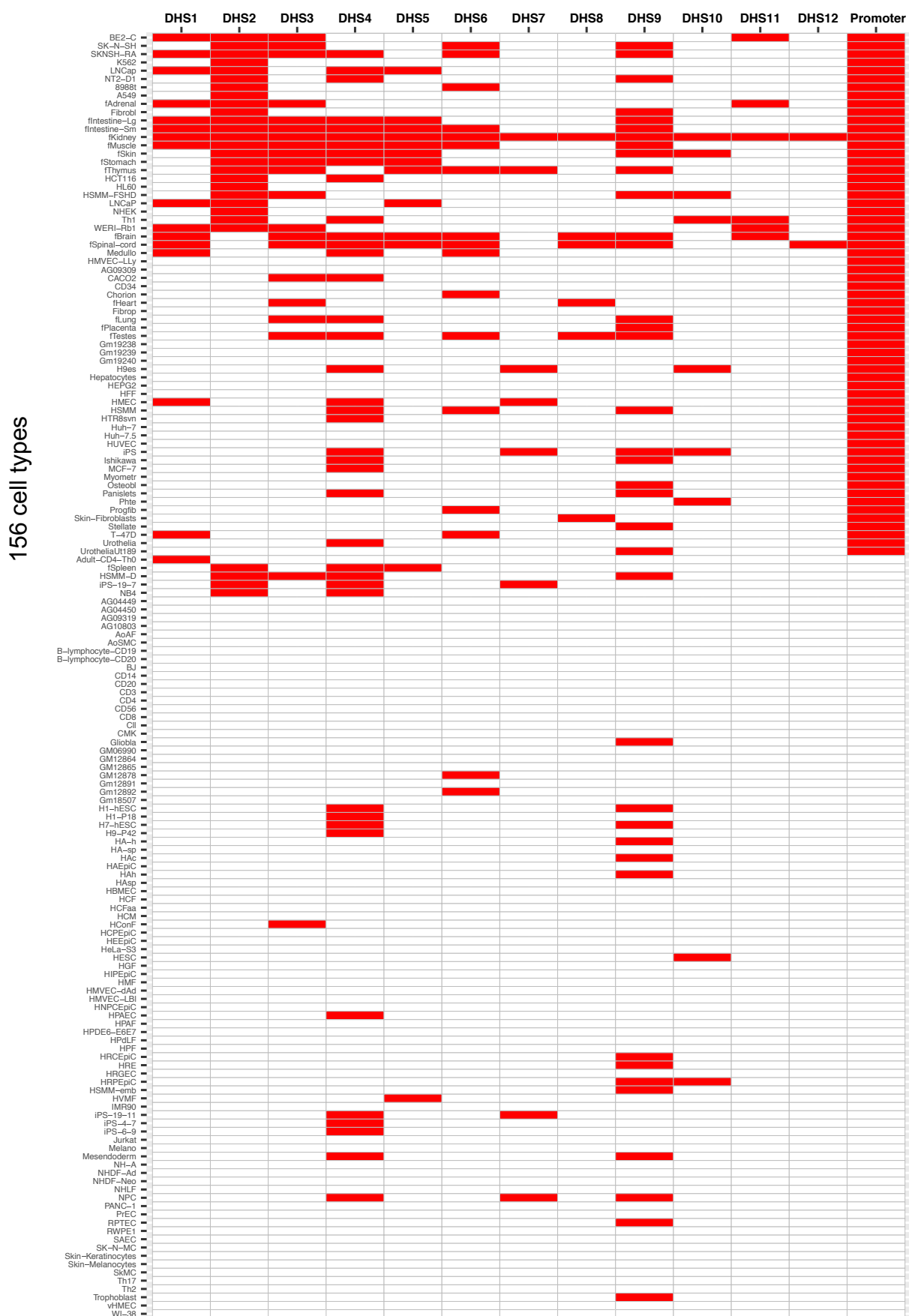

Supplement: S3 Fig — The 12 cis-regulatory regions were identified based on the correlation of the sequencing tag density between distal DHSs and proximal DHSs across different cell types within a human brain TAD. Shown here is a cell-type-specific DHS map for these 12 regions and ADRA2C promoter (columns). We combined DHS datasets from the ENCODE project and Roadmap Epigenomics Project, covering 156 cell types (rows). (PDF) [file pgen.1007311.s003.pdf]

## Supplementary Fig. 4

A

[illegible]

Supplementary Fig. 4

B

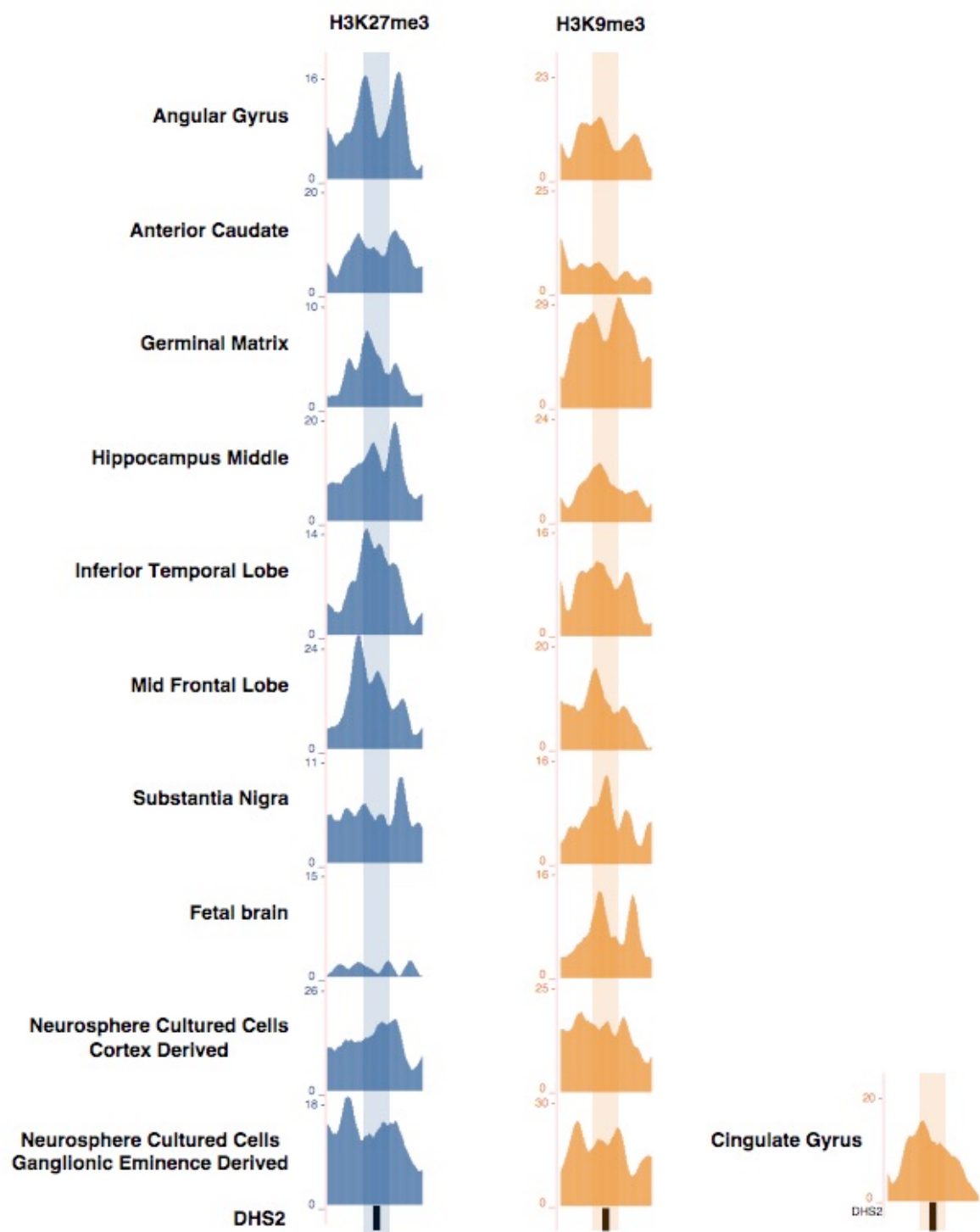

Supplement: S4 Fig — (A) Histone modification patterns of the 12 ADRA2C regulatory regions. ChIP-seq data for activating histone modifications (H3K4me1, H3K4me3, H3K9ac, H3K27ac, and H3K36me3) and repressive histone modifications (H3K9me3 and H3K27me3) in 11 brain tissues (Fetal brain, Germinal matrix, Neurosphere ganglionic eminence derived, Neurosphere cortex derived, Substantia nigra, Mid frontal lobe, Inferior temporal lobe, Hippocampus middle, Cingulate gyrus, Anterior caudate, and Angular gyrus) were obtained from the Roadmap Epigenomics project. Peak finding was performed by using HOMER. We obtained 14 fetal brain DHS datasets from the Roadmap Epigenomics project and 13 postnatal brain DHS datasets from the ENCODE project. They were merged into five categories (fetal brain, fetal spinal cord, neural progenitor cells, adult brain, and infant brain). The 12 ADRA2C regulatory regions were mapped to the histone modification peaks or DHSs. (B) ChIP-seq signals for H3K27me3 and H3K9me3 near DHS2 in the brain tissues shown in (A). (PDF) [file pgen.1007311.s004.pdf]

Supplementary Fig. 5

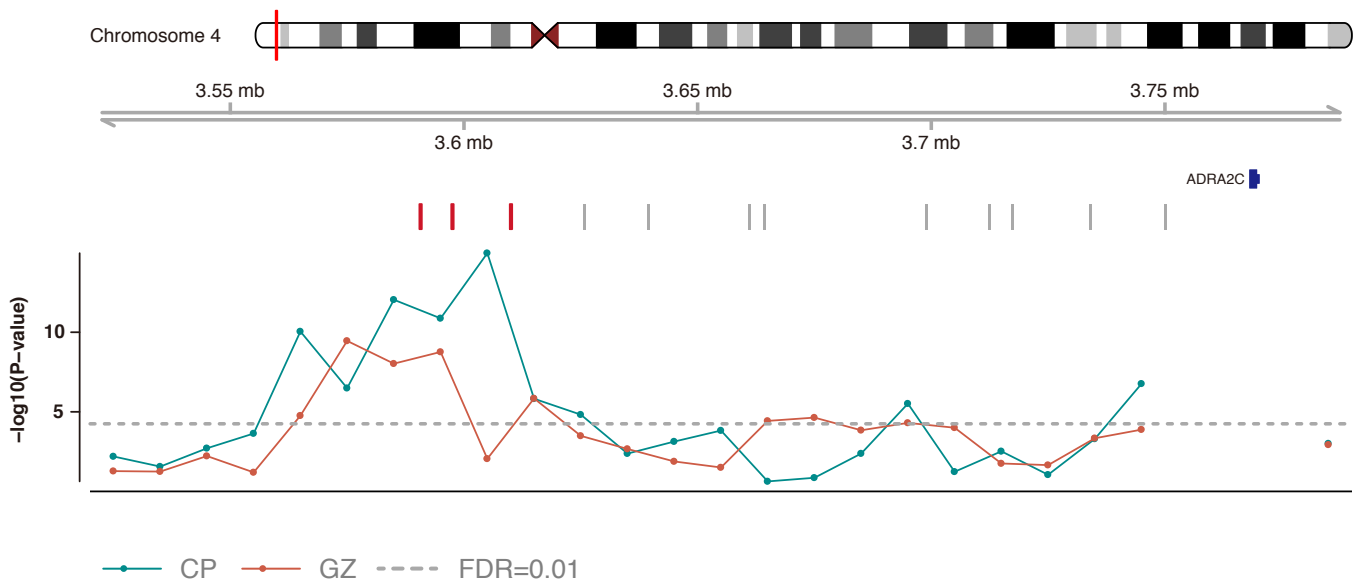

Supplement: S5 Fig — Statistical significance of its Hi-C interactions with the 12 distal regulatory regions (red and grey ticks below the chromosome ideogram and genome axis on the top) was measured for 10-kb bins using a background Hi-C interaction profile generated from random regions of the genome with matched GC content for gene promoters and was plotted as −log10[P value]. The green line is for the cortical and subcortical plate (CP) and the orange color line is for the germinal zone (GZ). The ADRA2C gene is marked in blue. The grey dotted line marks FDR = 0.01. (PDF) [file pgen.1007311.s005.pdf]

Supplementary Fig. 6

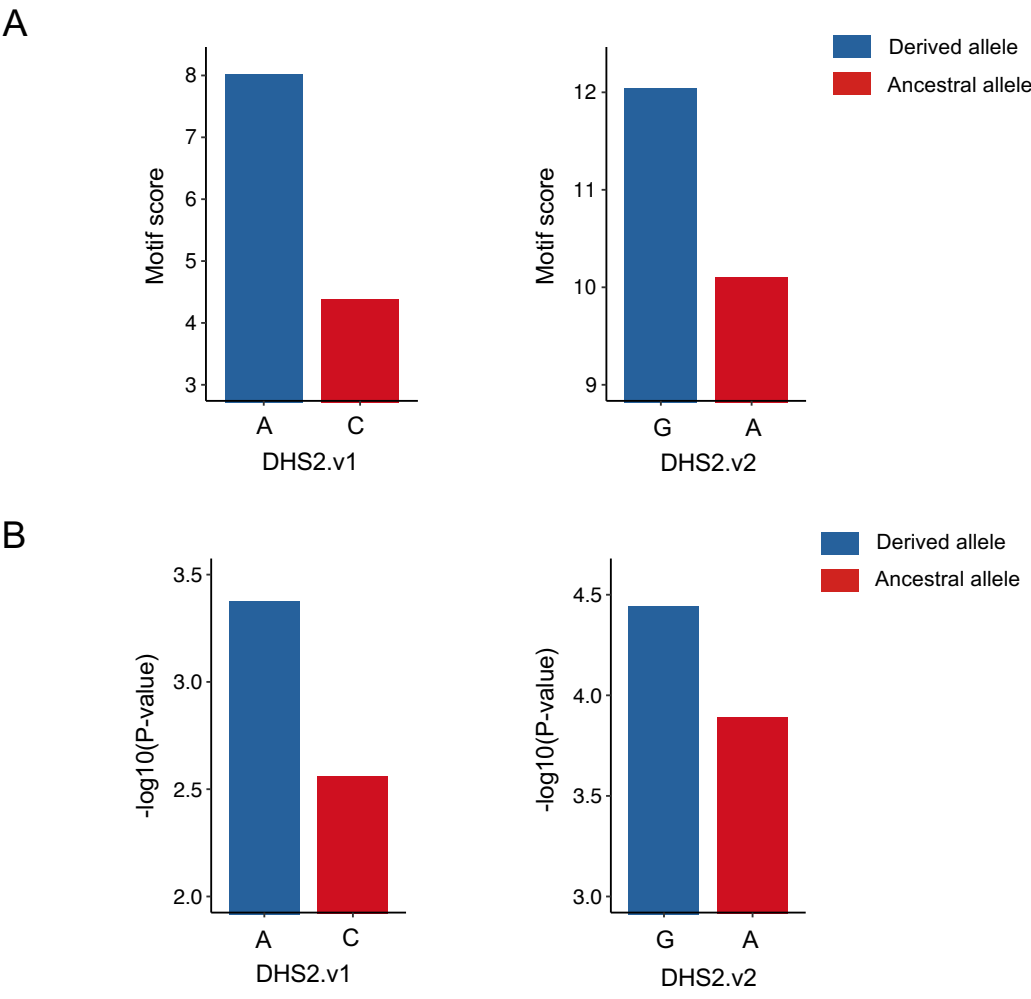

Supplement: S6 Fig — (A) Motif score derived from FIMO. (B) −log10[P value] of the FIMO motif score. (PDF) [file pgen.1007311.s006.pdf]

## Supplementary Fig. 7

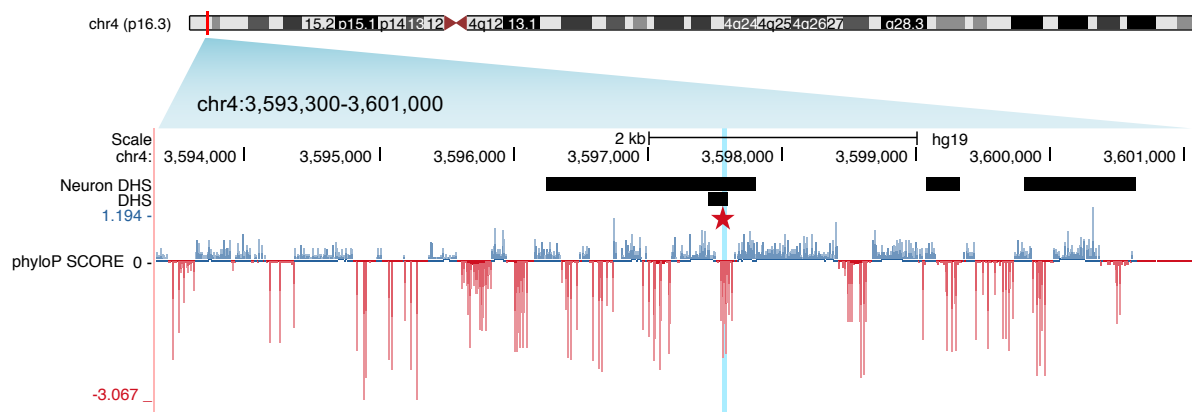

Supplement: S7 Fig — The location of the two NRSF motifs containing DHS2.v1 and DHS2.v2 is marked. The “Neuron DHS” track displays a union of DHSs in various neural cell lines and brain tissues, including BE(2)C, SKNSH-RA, SK-N-MC, NPC (H1 derived neuroprogenitor cells), NT2_D1, fetal brain, and fetal spinal cord, obtained from the ENCODE project and Roadmap Epigenomics project. We applied phyloP for the multiple alignments of primate genome sequences with “--subtree hg19-panTro2”, “--method LRT”, “--mode CONACC”, and “--wig-scores” as options. Negative values (red lines) indicate acceleration. (PDF) [file pgen.1007311.s007.pdf]

Supplementary Fig. 8

DHS2.v1

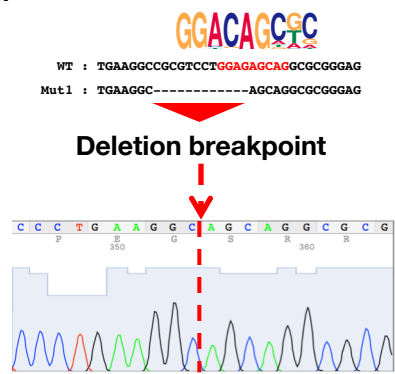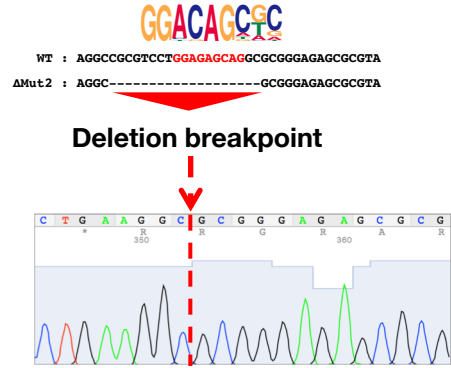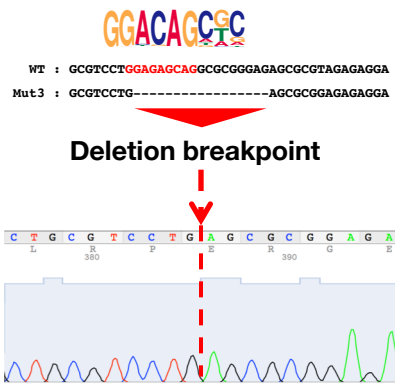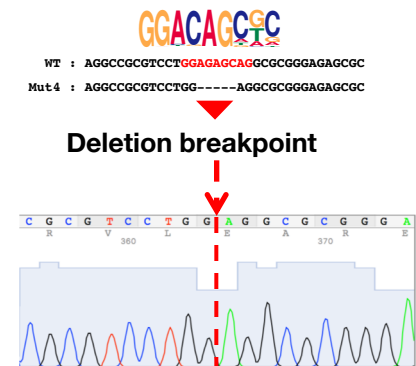

DHS2.v2

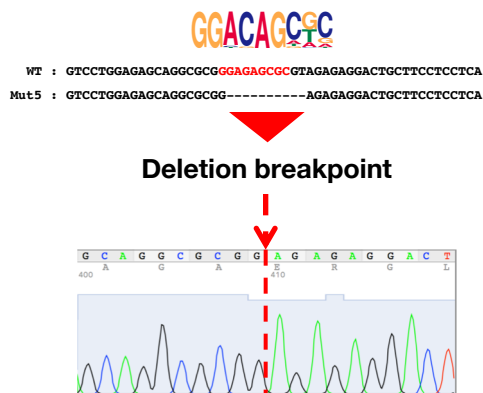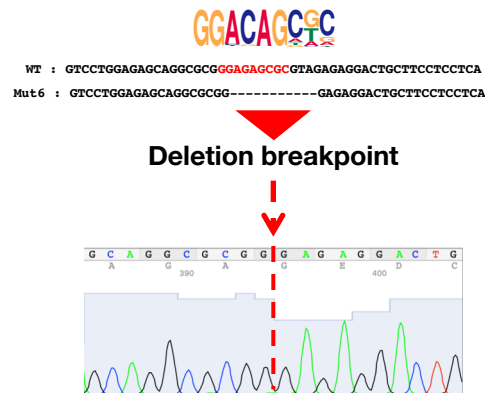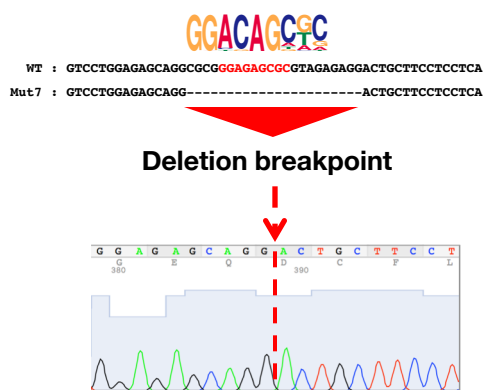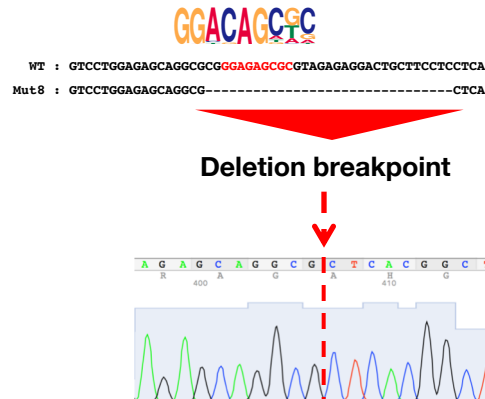

Supplement: S8 Fig — Deletion breakpoints generated by CRISPR/Cas9 for the four DHS2.v1 clones (upper) and the four DHS2.v2 clones (lower). The sequences of each PCR fragment were used to characterize the deletions. The NRSF binding motif, which was targeted for deletion, is marked in red. (PDF) [file pgen.1007311.s008.pdf]

Supplementary Fig. 9

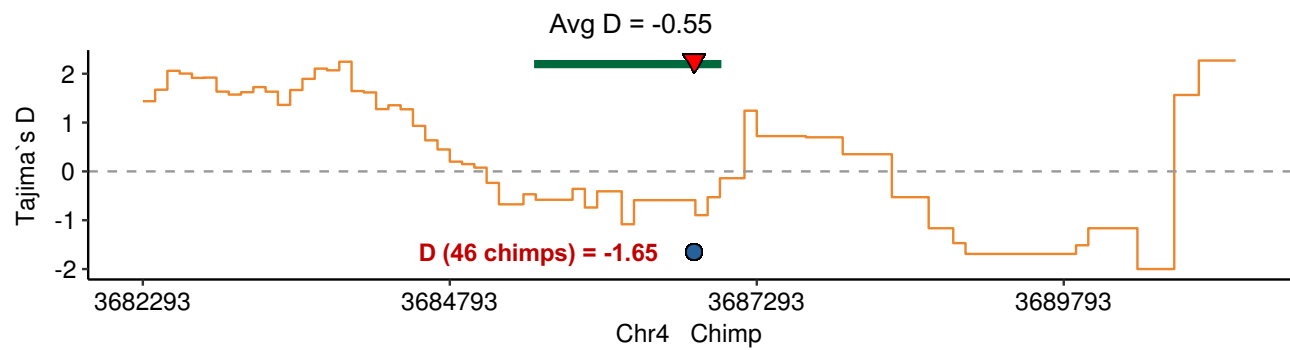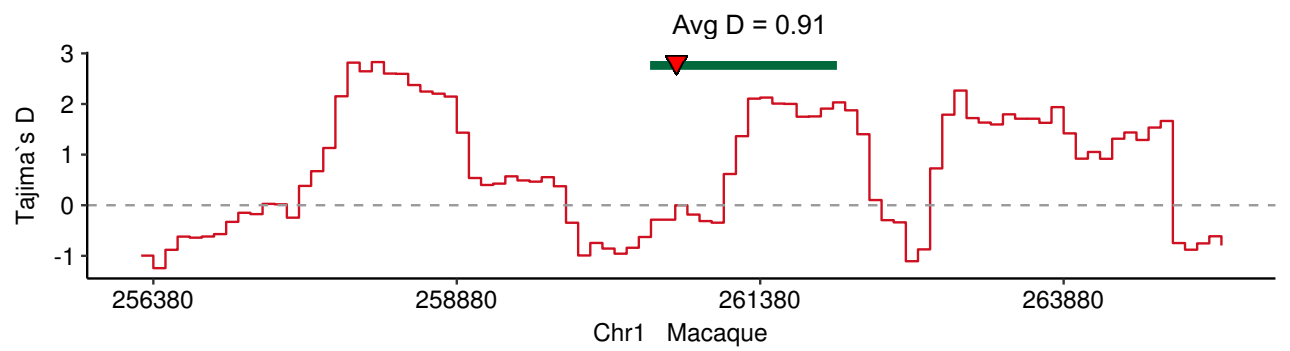

Supplement: S9 Fig — Tajima’s D for the DHS2 locus in chimpanzees (upper) and macaques (lower). D was calculated for the +/- 4.5 kb region using a 1-kb window with a step size of 100 bp. Negative values imply positive selection. The green bar is a neuronal DHS that encompasses the DHS2 SNPs (DHS2.v1 and DHS2.v2), whose location is marked by the red arrowhead. The blue dot in the chimpanzee plot indicates D for the 337-bp flanking sequences of the 46 chimpanzee samples. The average D for the neuronal DHS is shown. (PDF) [file pgen.1007311.s009.pdf]

Supplementary Fig. 10

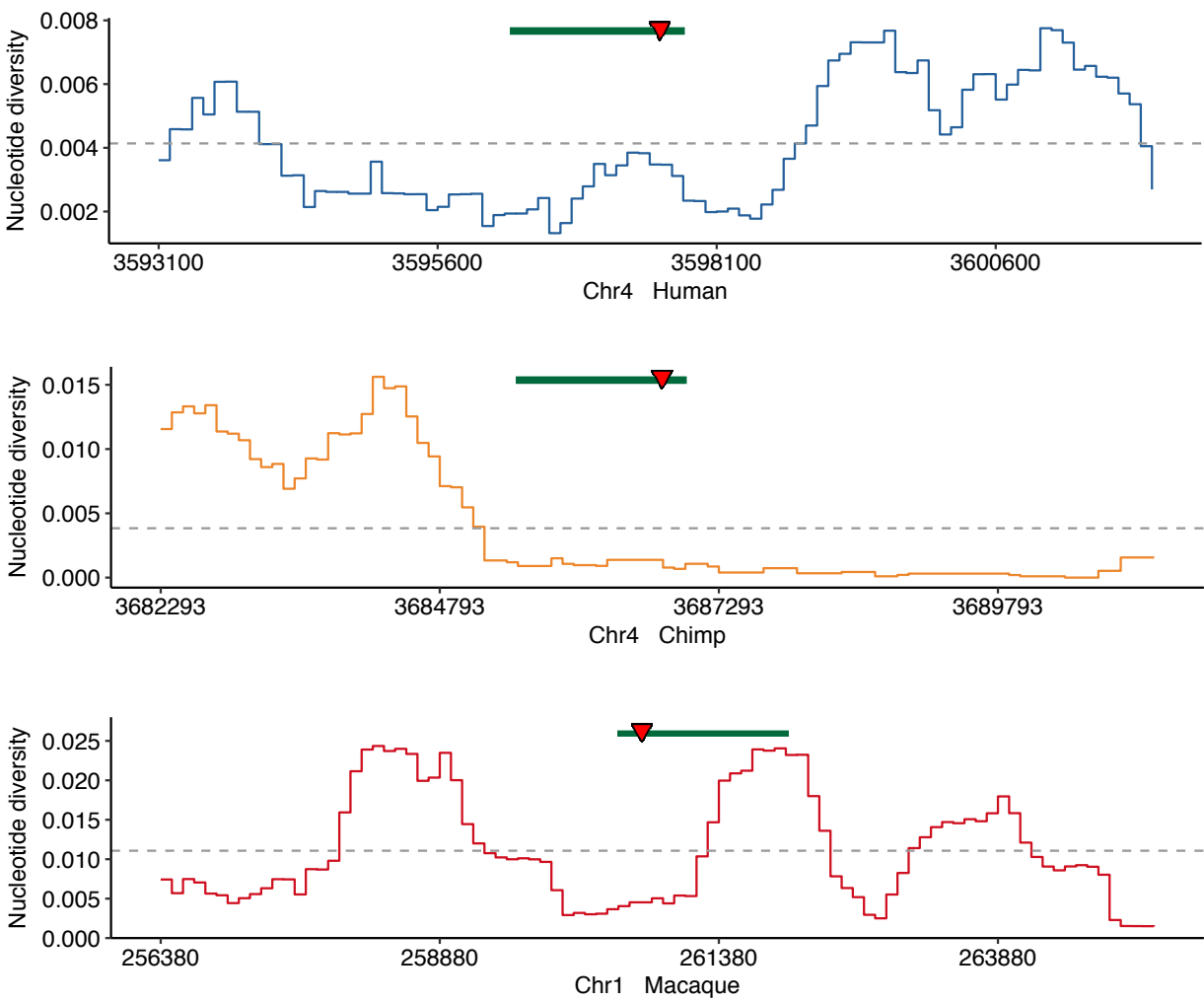

Supplement: S10 Fig — Nucleotide diversity for the DHS2 locus in humans (top), chimpanzees (middle), and macaques (bottom) whose whole-genome sequences were available. π was calculated for the +/- 4.5 kb region using a 1-kb window with a step size of 100 bp. Low nucleotide diversity is associated with positive selection. The green bar is a neuronal DHS that encompasses the DHS2 SNPs (DHS2.v1 and DHS2.v2), whose location is marked by the red arrowhead. The grey dotted horizontal lines mark the average diversity of the region. (PDF) [file pgen.1007311.s010.pdf]
