# Supplementary material for: Selection on the regulation of sympathetic nervous activity in humans and chimpanzees
Source: PLoS Genet. 2018 Apr 19;14(4):e1007311. doi: 10.1371/journal.pgen.1007311 (PMC5908061; doi:10.1371/journal.pgen.1007311)
Supplement: S11 Fig — A positive (or negative) iHS score means that haplotypes on the ancestral (or derived) allele background are longer compared to the derived (or ancestral) allele background. The last column of the below table shows the selected allele inferred according to the sign of the iHS score (A for positive iHS and D for negative iHS). The candidate variant for selection was highlighted. A possible scenario is that the ancestral haplotype acquired the derived sequence, T, before human-chimpanzee divergence at this position, which has been selected in the two species. This may be why the iHS results suggest selection for the alleles carried on the ancestral haplotype. (PDF) [file pgen.1007311.s011.pdf]

Supplementary Fig. 11

Position	Major allele	Minor allele	MAF	Multiz Alignments	iHS	Selected allele
chr4:3597315	G	T	0.230032	G,T,T,T,T	-1.0761987	G (D)
chr4:3597560	C	T	0.229034	C,C,C,C,C	2.2481296	C (A)
chr4:3597592	T	G	0.228834	T,G,G,G,G	-1.0705085	T (D)
chr4:3597627	G	T	0.228235	G,G,G,G,G	2.23083517	G (A)
**chr4:3597632**	**T**	**C**	**0.256989**	**T,T,C,C,C**	**3.37772303**	**T (A)**
chr4:3597723	A	G	0.230032	A,G,-,G,G	-1.0665686	A (D)
chr4:3597804	G	T	0.230032	G,T,T,T,T	-1.0696694	G (D)
chr4:3597843	G	A	0.205072	G,G,G,A,A	2.18660086	G (A)
chr4:3598032	G	C	0.230032	G,G,G,G,G	2.21454459	G (A)
chr4:3598161	C	T	0.230232	C,C,C,-,C	2.21574716	C (A)
chr4:3598593	T	C	0.232428	T,C,C,C,C	-1.02449	T (D)
chr4:3598709	G	A	0.232428	G,G,A,G,G	2.20515825	G (A)
chr4:3598829	C	G	0.232827	C,C,C,C,C	2.20911015	C (A)

MAF: Minor allele frequency

Multiz Alignments: Human, Chimpanzee, Gorilla, Orangutan, Macaque

D: Derived allele

A: Ancestral allele
