# Supplementary material for: Selection on the regulation of sympathetic nervous activity in humans and chimpanzees
Source: PLoS Genet. 2018 Apr 19;14(4):e1007311. doi: 10.1371/journal.pgen.1007311 (PMC5908061; doi:10.1371/journal.pgen.1007311)
Supplement: S1 Table — (PDF) [file pgen.1007311.s012.pdf]

Supplementary Table 1. Chromosomal coordinates of ADRA2C regulatory regions identified based on DNase I hypersensitivity

DHS ID	Chromosome	Start	End	Overlapping neural DHS	Neural DHS Start	Neural DHS End
DHS1	chr4	3590640	3590790	Y	3589805	3593208
DHS2	chr4	3597460	3597610	Y	3596244	3597804
DHS3	chr4	3609980	3610130	Y	3609350	3610275
DHS4	chr4	3625640	3625790	Y	3625312	3626157
DHS5	chr4	3639460	3639610	Y	3639185	3639690
DHS6	chr4	3660940	3661090	Y	3659430	3661285
DHS7	chr4	3664140	3664290	Y	3664020	3664252
DHS8	chr4	3698920	3699070	Y	3698802	3699109
DHS9	chr4	3712360	3712510	Y	3712158	3712701
DHS10	chr4	3717200	3717350	N	-	-
DHS11	chr4	3733900	3734050	Y	3733558	3734127
DHS12	chr4	3749980	3750130	Y	3749293	3750456
Promoter DHS	chr4	3768040	3768190	Y	3766562	3769841
