# Supplementary material for: Selection on the regulation of sympathetic nervous activity in humans and chimpanzees
Source: PLoS Genet. 2018 Apr 19;14(4):e1007311. doi: 10.1371/journal.pgen.1007311 (PMC5908061; doi:10.1371/journal.pgen.1007311)
Supplement: S2 Table — (PDF) [file pgen.1007311.s013.pdf]

Supplementary Table 2. Conservation or acceleration of ADRA2C regulatory sequences as estimated based on the likelihood ratio test of phyloP for the subtree of human and chimpanzee

DHS ID	Chromosome	Start	End	null_scale	alt_scale	alt_subscale	Log likelihood ratio	P value*
DHS1	chr4	3589805	3593208	1.22195	1.22245	0.98282	0.00452	0.92421
DHS2	chr4	3596244	3597804	1.49142	1.46818	1.42092	2.48091	-0.02591
DHS3	chr4	3609350	3610275	1.4942	1.37032	2.00596	7.02798	-0.00018
DHS4	chr4	3625312	3626157	1.3985	1.42282	0.63854	1.32054	0.10413
DHS5	chr4	3639185	3639690	1.39638	1.40729	0.90556	0.04772	0.75737
DHS6	chr4	3659430	3661285	1.59792	1.59824	0.9957	0.0004	0.97751
DHS7	chr4	3664020	3664252	2.26164	2.21885	1.11135	0.03683	-0.78607
DHS8	chr4	3698802	3699109	1.18605	1.27868	0.56238	0.51997	0.30784
DHS9	chr4	3712158	3712701	1.54323	1.56776	0.64661	0.84596	0.19335
DHS10	-	-	-	-	-	-	-	-
DHS11	chr4	3733558	3734127	1.32098	1.33055	0.75923	0.36341	0.39392
DHS12	chr4	3749293	3750456	1.5945	1.64108	0.70756	1.22061	0.11818

* Positive P values indicate evolutionary conservation and negative P values denote evolutionary acceleration
