# Supplementary material for: Selection on the regulation of sympathetic nervous activity in humans and chimpanzees
Source: PLoS Genet. 2018 Apr 19;14(4):e1007311. doi: 10.1371/journal.pgen.1007311 (PMC5908061; doi:10.1371/journal.pgen.1007311)
Supplement: S3 Table — (PDF) [file pgen.1007311.s014.pdf]

**Supplementary Table 3.** List of 10 unrelated chimpanzee samples whose genome data was used in this work. These samples were sequenced at the average sequencing coverage of 9.1.

| NCBI SRA Accession | Sample Name | Colony source of sample |
|---|---|---|
| ERR225036 | PtYO | Biomedical Primate Research Center |
| ERR225035 | PtSU | Biomedical Primate Research Center |
| ERR225034 | PtRN | Biomedical Primate Research Center |
| ERR225033 | PtRN | Biomedical Primate Research Center |
| ERR225032 | PtRG | Biomedical Primate Research Center |
| ERR225031 | PtPE | Biomedical Primate Research Center |
| ERR225030 | PtLI | Biomedical Primate Research Center |
| ERR225029 | PtLA | Biomedical Primate Research Center |
| ERR225028 | PtFR | Biomedical Primate Research Center |
| ERR225027 | PtAC | Biomedical Primate Research Center |
