# Supplementary material for: Selection on the regulation of sympathetic nervous activity in humans and chimpanzees
Source: PLoS Genet. 2018 Apr 19;14(4):e1007311. doi: 10.1371/journal.pgen.1007311 (PMC5908061; doi:10.1371/journal.pgen.1007311)
Supplement: S5 Table — (PDF) [file pgen.1007311.s016.pdf]

Supplementary Table 5. List of 46 genotyped chimpanzee samples.

No.	Sample ID	Source	Gender	Purchased From	Age
1	NS03622	Whole Blood	Female	Coriell Cell repositories	NA
2	NS03623	Whole Blood	Male	Coriell Cell repositories	14
3	NS03641	Whole Blood	Male	Coriell Cell repositories	32
4	NS03650	Whole Blood	Female	Coriell Cell repositories	11
5	NS03656	Whole Blood	Male	Coriell Cell repositories	14
6	NS03657	Whole Blood	Male	Coriell Cell repositories	36
7	NS03659	Whole Blood	Female	Coriell Cell repositories	10
8	NS03660	Whole Blood	Male	Coriell Cell repositories	46
9	S003624	Cell Culture (Fibroblast)	Male	Coriell Cell repositories	14
10	S003651	Cell Culture (Fibroblast)	Female	Coriell Cell repositories	11
11	S004920	Cell Culture (Fibroblast)	Male	Coriell Cell repositories	NA
12	S004933	Cell Culture (Fibroblast)	Female	Coriell Cell repositories	NA
13	S004971	Cell Culture (B-Lymphocyte)	Female	Coriell Cell repositories	NA
14	S005062	Cell Culture (B-Lymphocyte)	Male	Coriell Cell repositories	15
15	S005224	Cell Culture (B-Lymphocyte)	Female	Coriell Cell repositories	21
16	S005239	Cell Culture (B-Lymphocyte)	Female	Coriell Cell repositories	15
17	S005293	Cell Culture (B-Lymphocyte)	Female	Coriell Cell repositories	14
18	S005295	Cell Culture (B-Lymphocyte)	Female	Coriell Cell repositories	57
19	S005299	Cell Culture (B-Lymphocyte)	Male	Coriell Cell repositories	13
20	S005435	Cell Culture (B-Lymphocyte)	Female	Coriell Cell repositories	22
21	S005440	Cell Culture (B-Lymphocyte)	Male	Coriell Cell repositories	9
22	S005549	Cell Culture (B-Lymphocyte)	Female	Coriell Cell repositories	5
23	S005551	Cell Culture (B-Lymphocyte)	Female	Coriell Cell repositories	34
24	S005579	Cell Culture (B-Lymphocyte)	Female	Coriell Cell repositories	22
25	S005795	Cell Culture (Fibroblast)	Female	Coriell Cell repositories	26
26	S005803	Cell Culture (B-Lymphocyte)	Male	Coriell Cell repositories	20
27	S005804	Cell Culture (B-Lymphocyte)	Male	Coriell Cell repositories	13
28	S005823	Cell Culture (B-Lymphocyte)	Male	Coriell Cell repositories	10
29	S005837	Cell Culture (B-Lymphocyte)	Male	Coriell Cell repositories	16
30	S006003	Cell Culture (B-Lymphocyte)	Male	Coriell Cell repositories	14
31	S008842	Cell Culture (Fibroblast)	Male	Coriell Cell repositories	21
32	S008843	Cell Culture (Fibroblast)	Male	Coriell Cell repositories	23
33	S008887	Cell Culture (Fibroblast)	Male	Coriell Cell repositories	13
34	S008888	Cell Culture (Fibroblast)	Female	Coriell Cell repositories	NA
35	S008895	Cell Culture (B-Lymphocyte)	Female	Coriell Cell repositories	16

36	S008919	Cell Culture (Fibroblast)	Female	Coriell Cell repositories	10
37	S008933	Cell Culture (Fibroblast)	Female	Coriell Cell repositories	10
38	S008956	Cell Culture (Fibroblast)	Male	Coriell Cell repositories	17
39	S011081	Cell Culture (Fibroblast)	Female	Coriell Cell repositories	NA
40	S0336665	Cell Culture (Fibroblast)	Female	Coriell Cell repositories	1 Day Old
41	C0389	Liver Tissue	Male	Yerkes National Primate Research Center	NA
42	C0462	Liver Tissue	Female	Yerkes National Primate Research Center	NA
43	C0554	Liver Tissue	Female	Yerkes National Primate Research Center	NA
44	C0568	Liver Tissue	Female	Yerkes National Primate Research Center	NA
45	C0591	Liver Tissue	Male	Yerkes National Primate Research Center	NA
46	C0593	Liver Tissue	Male	Yerkes National Primate Research Center	NA
