# Supplementary material for: Selection on the regulation of sympathetic nervous activity in humans and chimpanzees
Source: PLoS Genet. 2018 Apr 19;14(4):e1007311. doi: 10.1371/journal.pgen.1007311 (PMC5908061; doi:10.1371/journal.pgen.1007311)
Supplement: S6 Table — (PDF) [file pgen.1007311.s017.pdf]

Supplementary Table 1. Chromosomal coordinates of ADRA2C regulatory regions identified based on DNase I hypersensitivity

DHS ID	Chromosome	Start	End	Overlapping neural DHS	Neural DHS Start	Neural DHS End
DHS1	chr4	3590640	3590790	Y	3589805	3593208
DHS2	chr4	3597460	3597610	Y	3596244	3597804
DHS3	chr4	3609980	3610130	Y	3609350	3610275
DHS4	chr4	3625640	3625790	Y	3625312	3626157
DHS5	chr4	3639460	3639610	Y	3639185	3639690
DHS6	chr4	3660940	3661090	Y	3659430	3661285
DHS7	chr4	3664140	3664290	Y	3664020	3664252
DHS8	chr4	3698920	3699070	Y	3698802	3699109
DHS9	chr4	3712360	3712510	Y	3712158	3712701
DHS10	chr4	3717200	3717350	N	-	-
DHS11	chr4	3733900	3734050	Y	3733558	3734127
DHS12	chr4	3749980	3750130	Y	3749293	3750456
Promoter DHS	chr4	3768040	3768190	Y	3766562	3769841

Supplementary Table 2. Conservation or acceleration of ADRA2C regulatory sequences as estimated based on the likelihood ratio test of phyloP for the subtree of human and chimpanzee

DHS ID	Chromosome	Start	End	null_scale	alt_scale	alt_subscale	Log likelihood ratio	P value*
DHS1	chr4	3589805	3593208	1.22195	1.22245	0.98282	0.00452	0.92421
DHS2	chr4	3596244	3597804	1.49142	1.46818	1.42092	2.48091	-0.02591
DHS3	chr4	3609350	3610275	1.4942	1.37032	2.00596	7.02798	-0.00018
DHS4	chr4	3625312	3626157	1.3985	1.42282	0.63854	1.32054	0.10413
DHS5	chr4	3639185	3639690	1.39638	1.40729	0.90556	0.04772	0.75737
DHS6	chr4	3659430	3661285	1.59792	1.59824	0.9957	0.0004	0.97751
DHS7	chr4	3664020	3664252	2.26164	2.21885	1.11135	0.03683	-0.78607
DHS8	chr4	3698802	3699109	1.18605	1.27868	0.56238	0.51997	0.30784
DHS9	chr4	3712158	3712701	1.54323	1.56776	0.64661	0.84596	0.19335
DHS10	-	-	-	-	-	-	-	-
DHS11	chr4	3733558	3734127	1.32098	1.33055	0.75923	0.36341	0.39392
DHS12	chr4	3749293	3750456	1.5945	1.64108	0.70756	1.22061	0.11818

* Positive P values indicate evolutionary conservation and negative P values denote evolutionary acceleration

Supplementary Table 3. List of 10 unrelated chimpanzee samples whose genome data was used in this work. These samples were sequenced at the average sequencing coverage of 9.1.

NCBI SRA Accession	Sample Name	Colony source of sample
ERR225036	PtYO	Biomedical Primate Research Center
ERR225035	PtSU	Biomedical Primate Research Center
ERR225034	PtRN	Biomedical Primate Research Center
ERR225033	PtRN	Biomedical Primate Research Center
ERR225032	PtRG	Biomedical Primate Research Center
ERR225031	PtPE	Biomedical Primate Research Center
ERR225030	PtLI	Biomedical Primate Research Center
ERR225029	PtLA	Biomedical Primate Research Center
ERR225028	PtFR	Biomedical Primate Research Center
ERR225027	PtAC	Biomedical Primate Research Center

Supplementary Table 4. List of 108 unrelated rhesus macaque samples whole genome data was used in this work

NCBI SRA Accession	Sample Name	Colony source of sample	Ancestry	Sex	Sequence Coverage
SAMN03264739	MMUL.CH-36390	California National Primate Research Center	Chinese	F	11.5
SAMN03264740	MMUL.CH-36394	California National Primate Research Center	Chinese	F	8.6
SAMN03264732	MMUL.IN-36332	California National Primate Research Center	Indian	M	9.7
SAMN03264736	MMUL.IN-36371	California National Primate Research Center	Indian	F	10.7
SAMN03264738	MMUL.CH-36389	California National Primate Research Center	Chinese	M	9.4
SAMN03264734	MMUL.IN-36357	California National Primate Research Center	Indian	F	10.3
SAMN03264735	MMUL.IN-36359	California National Primate Research Center	Indian	F	9.8
SAMN03264737	MMUL.IN-36374	California National Primate Research Center	Indian	F	9.9
SAMN03264733	MMUL.IN-36355	California National Primate Research Center	Indian	F	10.8
SAMN03264762	MMUL.IN-36460	Caribbean Primate Research Center	Indian	F	9.2
SAMN03264763	MMUL.IN-36467	Caribbean Primate Research Center	Indian	F	8.4
SAMN03264767	MMUL.IN-36477	Caribbean Primate Research Center	Indian	F	9
SAMN03264768	MMUL.IN-36476	Caribbean Primate Research Center	Indian	F	8.3
SAMN03264775	MMUL.IN-36474	Caribbean Primate Research Center	Indian	M	8.7
SAMN03264779	MMUL.IN-36466	Caribbean Primate Research Center	Indian	F	7.9
SAMN03264676	MMUL.IN-35250	New England Primate Research Center	Indian	F	7.7
SAMN03264677	MMUL.IN-35252	New England Primate Research Center	Indian	F	11
SAMN03264678	MMUL.IN-35253	New England Primate Research Center	Indian	F	7.9
SAMN03264679	MMUL.IN-35254	New England Primate Research Center	Indian	M	7
SAMN03264681	MMUL.IN-35256	New England Primate Research Center	Indian	F	10.3
SAMN03264683	MMUL.IN-35259	New England Primate Research Center	Indian	F	10.3
SAMN03264695	MMUL.IN-35717	Oregon National Primate Research Center	Indian	F	9.6
SAMN03264696	MMUL.IN-35718	Oregon National Primate Research Center	Indian	F	11.3
SAMN03264697	MMUL.IN-35722	Oregon National Primate Research Center	Indian	F	11.6
SAMN03264699	MMUL.IN-35724	Oregon National Primate Research Center	Indian	F	9.4
SAMN03264700	MMUL.IN-35728	Oregon National Primate Research Center	Indian	F	8.5
SAMN03264702	MMUL.IN-35730	Oregon National Primate Research Center	Indian	F	11.4
SAMN03264703	MMUL.IN-35732	Oregon National Primate Research Center	Indian	F	8.4
SAMN03264725	MMUL.IN-35969	Southwest National Primate Research Center	Indian	F	10.2
SAMN03264726	MMUL.IN-35972	Southwest National Primate Research Center	Indian	M	7.3
SAMN03264727	MMUL.IN-35975	Southwest National Primate Research Center	Indian	F	8.4
SAMN03264716	MMUL.IN-35895	Tulane National Primate Research Center	Indian	F	10.6
SAMN03264718	MMUL.IN-35907	Tulane National Primate Research Center	Indian	M	8.6

SAMN03264719	MMUL.IN-35916	Tulane National Primate Research Center	Indian	F	10.9
SAMN03264721	MMUL.IN-35921	Tulane National Primate Research Center	Indian	F	10
SAMN03264722	MMUL.IN-35923	Tulane National Primate Research Center	Indian	F	7.2
SAMN03264724	MMUL.IN-35957	Tulane National Primate Research Center	Indian	F	9.7
SAMN03264715	MMUL.IN-35883	Tulane National Primate Research Center	Indian	M	8
SAMN03264717	MMUL.IN-35902	Tulane National Primate Research Center	Indian	M	7.7
SAMN03264720	MMUL.IN-35919	Tulane National Primate Research Center	Indian	F	7.5
SAMN03264605	MMUL.IN-28499	Wisconsin National Primate Research Center	Indian	M	11.3
SAMN03264606	MMUL.IN-28500	Wisconsin National Primate Research Center	Indian	F	9.5
SAMN03264607	MMUL.IN-28507	Wisconsin National Primate Research Center	Indian	M	10.5
SAMN03264608	MMUL.IN-28518	Wisconsin National Primate Research Center	Indian	F	10.3
SAMN03264609	MMUL.IN-28535	Wisconsin National Primate Research Center	Indian	M	7.9
SAMN03264610	MMUL.IN-28555	Wisconsin National Primate Research Center	Indian	M	7.6
SAMN03264618	MMUL.IN-30423	Wisconsin National Primate Research Center	Indian	F	9.6
SAMN03264619	MMUL.IN-30424	Wisconsin National Primate Research Center	Indian	M	10.6
SAMN03264685	MMUL.IN-35490	Yerkes National Primate Research Center	Indian	F	11.4
SAMN03264689	MMUL.IN-35496	Yerkes National Primate Research Center	Indian	F	11.5
SAMN03264694	MMUL.IN-35502	Yerkes National Primate Research Center	Indian	F	8.9
SAMN03264597	MMUL.IN-18277	Yerkes National Primate Research Center	Indian	F	35.8
SAMN03264598	MMUL.IN-19466	Yerkes National Primate Research Center	Indian	M	36.2
SAMN03264600	MMUL.IN-24898	Wisconsin National Primate Research Center	Indian	F	39.4
SAMN03264613	MMUL.IN-30119	Wisconsin National Primate Research Center	Indian	F	35.9
SAMN03264614	MMUL.IN-30136	Wisconsin National Primate Research Center	Indian	M	29.9
SAMN03264620	MMUL.IN-31505	Wisconsin National Primate Research Center	Indian	F	36.5
SAMN03264621	MMUL.IN-32510	Wisconsin National Primate Research Center	Indian	F	25.6
SAMN03264623	MMUL.IN-32754	Wisconsin National Primate Research Center	Indian	F	27.7
SAMN03264629	MMUL.IN-34600	New England Primate Research Center	Indian	M	32.1
SAMN03264630	MMUL.IN-34602	New England Primate Research Center	Indian	M	35
SAMN03264635	MMUL.IN-34762	Yerkes National Primate Research Center	Indian	F	38.2
SAMN03264639	MMUL.IN-34770	Yerkes National Primate Research Center	Indian	F	37.3
SAMN03264641	MMUL.IN-35044	Oregon National Primate Research Center	Indian	M	36.2
SAMN03264642	MMUL.IN-35045	Oregon National Primate Research Center	Indian	M	60.7
SAMN03264643	MMUL.IN-35046	Oregon National Primate Research Center	Indian	M	34.2
SAMN03264645	MMUL.IN-35048	Oregon National Primate Research Center	Indian	M	40
SAMN03264646	MMUL.IN-35049	Oregon National Primate Research Center	Indian	M	42.7
SAMN03264649	MMUL.IN-35055	Oregon National Primate Research Center	Indian	F	32.6
SAMN03264650	MMUL.IN-35059	Oregon National Primate Research Center	Indian	M	37.2

SAMN03264651	MMUL.IN-35060	Oregon National Primate Research Center	Indian	M	41.2
SAMN03264652	MMUL.IN-35061	Oregon National Primate Research Center	Indian	M	40.8
SAMN03264653	MMUL.CH-35082	California National Primate Research Center	Chinese	F	45.7
SAMN03264658	MMUL.IN-35087	California National Primate Research Center	Indian	F	30.3
SAMN03264666	MMUL.IN-35095	California National Primate Research Center	Indian	M	36.1
SAMN03264667	MMUL.IN-35096	California National Primate Research Center	Indian	F	35.8
SAMN03264672	MMUL.IN-35154	New England Primate Research Center	Indian	M	33.5
SAMN03264674	MMUL.IN-35162	New England Primate Research Center	Indian	M	32.5
SAMN03264705	MMUL.IN-35864	Tulane National Primate Research Center	Indian	M	32.7
SAMN03264706	MMUL.IN-35865	Tulane National Primate Research Center	Indian	M	41
SAMN03264707	MMUL.IN-35866	Tulane National Primate Research Center	Indian	F	41
SAMN03264708	MMUL.IN-35868	Tulane National Primate Research Center	Indian	F	34.7
SAMN03264709	MMUL.IN-35871	Tulane National Primate Research Center	Indian	M	39.1
SAMN03264710	MMUL.IN-35872	Tulane National Primate Research Center	Indian	F	39.2
SAMN03264711	MMUL.IN-35873	Tulane National Primate Research Center	Indian	M	42.7
SAMN03264712	MMUL.IN-35874	Tulane National Primate Research Center	Indian	M	37.8
SAMN03264728	MMUL.IN-35976	Southwest National Primate Research Center	Indian	F	41.1
SAMN03264729	MMUL.IN-35990	Southwest National Primate Research Center	Indian	M	38.2
SAMN03264730	MMUL.CH-36013	California National Primate Research Center	Chinese	F	41
SAMN03264761	MMUL.IN-39345	California National Primate Research Center	Indian	F	42
SAMN03083651	MMUL.IN-36468	Caribbean Primate Research Center	Indian	F	40.4
SAMN03264769	MMUL.IN-36461	Caribbean Primate Research Center	Indian	M	41.1
SAMN03264770	MMUL.IN-36462	Caribbean Primate Research Center	Indian	M	38.2
SAMN03264771	MMUL.IN-36463	Caribbean Primate Research Center	Indian	M	39.1
SAMN03264774	MMUL.IN-36473	Caribbean Primate Research Center	Indian	M	42.3
SAMN03264780	MMUL.IN-36471	Caribbean Primate Research Center	Indian	F	40.9
SAMN03264781	MMUL.IN-36470	Caribbean Primate Research Center	Indian	F	40.3
SAMN03264764	MMUL.IN-11414-01b	Wisconsin National Primate Research Center	Indian	F	34.5
SAMN03264765	MMUL.IN-11433-07b	Wisconsin National Primate Research Center	Indian	F	53.3
SAMN03264743	MMUL.IN-37732	Wisconsin National Primate Research Center	Indian	F	44
SAMN03264744	MMUL.IN-37733	Wisconsin National Primate Research Center	Indian	F	42.5
SAMN03264746	MMUL.IN-37735	Wisconsin National Primate Research Center	Indian	M	41.3
SAMN03264749	MMUL.IN-37738	Wisconsin National Primate Research Center	Indian	F	38.8
SAMN03264750	MMUL.IN-37739	Wisconsin National Primate Research Center	Indian	M	40.7
SAMN03264751	MMUL.IN-37740	Wisconsin National Primate Research Center	Indian	M	41.6
SAMN03264756	MMUL.IN-37745	Wisconsin National Primate Research Center	Indian	F	39.6
SAMN03264757	MMUL.IN-37746	Wisconsin National Primate Research Center	Indian	F	38.6

SAMN03264760	MMUL.CH-37950	Wild Caught	Chinese	F	29.6

Supplementary Table 5. List of 46 genotyped chimpanzee samples.

No.	Sample ID	Source	Gender	Purchased From	Age
1	NS03622	Whole Blood	Female	Coriell Cell repositories	NA
2	NS03623	Whole Blood	Male	Coriell Cell repositories	14
3	NS03641	Whole Blood	Male	Coriell Cell repositories	32
4	NS03650	Whole Blood	Female	Coriell Cell repositories	11
5	NS03656	Whole Blood	Male	Coriell Cell repositories	14
6	NS03657	Whole Blood	Male	Coriell Cell repositories	36
7	NS03659	Whole Blood	Female	Coriell Cell repositories	10
8	NS03660	Whole Blood	Male	Coriell Cell repositories	46
9	S003624	Cell Culture (Fibroblast)	Male	Coriell Cell repositories	14
10	S003651	Cell Culture (Fibroblast)	Female	Coriell Cell repositories	11
11	S004920	Cell Culture (Fibroblast)	Male	Coriell Cell repositories	NA
12	S004933	Cell Culture (Fibroblast)	Female	Coriell Cell repositories	NA
13	S004971	Cell Culture (B-Lymphocyte)	Female	Coriell Cell repositories	NA
14	S005062	Cell Culture (B-Lymphocyte)	Male	Coriell Cell repositories	15
15	S005224	Cell Culture (B-Lymphocyte)	Female	Coriell Cell repositories	21
16	S005239	Cell Culture (B-Lymphocyte)	Female	Coriell Cell repositories	15
17	S005293	Cell Culture (B-Lymphocyte)	Female	Coriell Cell repositories	14
18	S005295	Cell Culture (B-Lymphocyte)	Female	Coriell Cell repositories	57
19	S005299	Cell Culture (B-Lymphocyte)	Male	Coriell Cell repositories	13
20	S005435	Cell Culture (B-Lymphocyte)	Female	Coriell Cell repositories	22
21	S005440	Cell Culture (B-Lymphocyte)	Male	Coriell Cell repositories	9
22	S005549	Cell Culture (B-Lymphocyte)	Female	Coriell Cell repositories	5
23	S005551	Cell Culture (B-Lymphocyte)	Female	Coriell Cell repositories	34
24	S005579	Cell Culture (B-Lymphocyte)	Female	Coriell Cell repositories	22
25	S005795	Cell Culture (Fibroblast)	Female	Coriell Cell repositories	26
26	S005803	Cell Culture (B-Lymphocyte)	Male	Coriell Cell repositories	20
27	S005804	Cell Culture (B-Lymphocyte)	Male	Coriell Cell repositories	13
28	S005823	Cell Culture (B-Lymphocyte)	Male	Coriell Cell repositories	10
29	S005837	Cell Culture (B-Lymphocyte)	Male	Coriell Cell repositories	16
30	S006003	Cell Culture (B-Lymphocyte)	Male	Coriell Cell repositories	14
31	S008842	Cell Culture (Fibroblast)	Male	Coriell Cell repositories	21
32	S008843	Cell Culture (Fibroblast)	Male	Coriell Cell repositories	23
33	S008887	Cell Culture (Fibroblast)	Male	Coriell Cell repositories	13
34	S008888	Cell Culture (Fibroblast)	Female	Coriell Cell repositories	NA
35	S008895	Cell Culture (B-Lymphocyte)	Female	Coriell Cell repositories	16

36	S008919	Cell Culture (Fibroblast)	Female	Coriell Cell repositories	10
37	S008933	Cell Culture (Fibroblast)	Female	Coriell Cell repositories	10
38	S008956	Cell Culture (Fibroblast)	Male	Coriell Cell repositories	17
39	S011081	Cell Culture (Fibroblast)	Female	Coriell Cell repositories	NA
40	S0336665	Cell Culture (Fibroblast)	Female	Coriell Cell repositories	1 Day Old
41	C0389	Liver Tissue	Male	Yerkes National Primate Research Center	NA
42	C0462	Liver Tissue	Female	Yerkes National Primate Research Center	NA
43	C0554	Liver Tissue	Female	Yerkes National Primate Research Center	NA
44	C0568	Liver Tissue	Female	Yerkes National Primate Research Center	NA
45	C0591	Liver Tissue	Male	Yerkes National Primate Research Center	NA
46	C0593	Liver Tissue	Male	Yerkes National Primate Research Center	NA

Supplementary Table 6. CRISPR/Cas9 single guide RNA (sgRNA) sequences and PCR primers used in this study.

**Primers for human genomic DNA PCR**		
Target site	Orientation	Sequence
DHS2	Forward	5′- GACTGACCGAGCTCAGTGAGA -3′
	Reverse	5′- TCGGAGAGACACGCAGACAT -3′

**Primers for chimpanzee genomic DNA PCR**		
Target site	Orientation	Sequence
DHS2	Forward	5′- GCTCAGTATCCAGAAGGACCTTC -3′
	Reverse	5′- ATCACCTGAAGCAGCTGTCTC -3′

**sgRNA sequences**		
Target site	Orientation	Sequence
DHS2.v1	Forward	5′- CACCGAAGGCCGCGTCCTGGAGAGC -3′
	Reverse	5′- AAACGCTCTCCAGGACGCGGCCTTC -3′
DHS2.v2	Forward	5′- CACCGGCGCGGGAGAGCGCGTAGAG -3′
	Reverse	5′- AAACCTCTACGCGCTCTCCCGCGCC -3′

**Primers for RT-qPCR**		
Target site	Orientation	Sequence
ADRA2C	Forward	5′- GCCTCAACGACGAGACCTG -3′
	Reverse	5′- CCCAGCCCGTTTTCGGTAG -3′
GAPDH	Forward	5′- GAGTCAACGGATTTGGTCGT -3′
	Reverse	5′- GACAAGCTTCCCGTTCTCAG -3′
